# Conformal 3D Li/Li_13_Sn_5_ Scaffolds Anodes for High‐Areal Energy Density Flexible Lithium Metal Batteries

**DOI:** 10.1002/advs.202309254

**Published:** 2024-02-07

**Authors:** Xiaomei Huo, Xin Gong, Yuhang Liu, Yonghui Yan, Zhuzhu Du, Wei Ai

**Affiliations:** ^1^ Frontiers Science Center for Flexible Electronics & Xi'an Institute of Flexible Electronics Northwestern Polytechnical University Xi'an 710072 China

**Keywords:** lithium metal, Li_13_Sn_5_, flexible batteries, depth of discharge, areal energy density

## Abstract

Achieving a high depth of discharge (DOD) in lithium metal anodes (LMAs) is crucial for developing high areal energy density batteries suitable for wearable electronics. Yet, the persistent growth of dendrites compromises battery performance, and the significant lithium consumption during pre‐lithiation obstructs their broad application. Herein, A flexible 3D Li_13_Sn_5_ scaffold is designed by allowing molten lithium to infiltrate carbon cloth adorned with SnO_2_ nanocrystals. This design markedly curbs the troublesome dendrite growth, thanks to the uniform electric field distribution and swift Li^+^ diffusion dynamics. Additionally, with a minimal SnO_2_ nanocrystals loading (2 wt.%), only 0.6 wt.% of lithium is consumed during pre‐lithiation. Insights from in situ optical microscope observations and COMSOL simulations reveal that lithium remains securely anchored within the scaffold, a result of the rapid mass/charge transfer and uniform electric field distribution. Consequently, this electrode achieves a remarkable DOD of 87.1% at 10 mA cm^−2^ for 40 mAh cm^−2^. Notably, when coupled with a polysulfide cathode, the constructed flexible Li/Li_13_Sn_5_@CC||Li_2_S_6_/SnO_2_@CC pouch cell delivers a high‐areal capacity of 5.04 mAh cm^−2^ and an impressive areal‐energy density of 10.6 mWh cm^−2^. The findings pave the way toward the development of high‐performance LMAs, ideal for long‐lasting wearable electronics.

## Graphical abstract

A novel flexible 3D Li_13_Sn_5_ scaffold for lithium metal batteries boasts exceptional efficiency with only 0.6 wt.% lithium used in pre‐lithiation. This design effectively curtails dendrite growth and lithium volume expansion, enabling the pouch cell to deliver a high areal capacity of 5.04 mAh cm^−2^ and a remarkable energy density of 10.6 mWh cm^−2^.

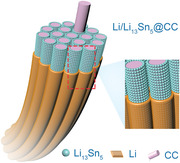

## 1 Introduction

The surge in demand for mobile electronics underscores the immediate need for portable batteries boasting high areal energy/power density.^[^ ^1^ ^]^ Among potential candidates, the Li metal anode (LMA) distinguishes itself, thanks to its impressive theoretical capacity (3860 mAh g^−1^) and low redox potential (−3.04 V vs the standard hydrogen electrode).^[^ ^2^ ^]^ Especially when paired with emerging cathodes like sulfur or oxygen, LMAs can notably amplify a battery's energy density.^[^ ^3^ ^]^ Nonetheless, LMAs' widespread adoption is hampered by challenges like limited rate capability,^[^ ^4^ ^]^ subpar depth of discharge (DOD),^[^ ^5^ ^]^ and safety risks.^[^ ^6^ ^]^ It is worth noting that achieving a high DOD is essential for batteries with a dense energy profile.^[^ ^7^ ^]^ The sluggish Li^+^ diffusion kinetics in bulk Li metal (5.69 × 10^−11^ cm^2^ S^−1^) compromises LMAs' rate performance, while the vexing issue of Li dendrite growth diminishes the DOD, even posing risks of catastrophic battery failures.^[^ ^8^ ^]^

A variety of strategies have so far been explored to address these concerns, including surface alterations, electrolyte tweaking, and introducing high‐efficiency Li storage hosts.^[^ ^9^ ^]^ Notably, compared to planar electrode structures, infusing metallic Li into a 3D host emerges as a favorable approach for boosting DOD,^[^ ^10^ ^]^ as such hosts can curtail dendrite formation through uniform electric field distribution and offering ample volume for metallic Li accommodation.^[^ ^11^ ^]^ However, owing to the limited research, current electrodes mainly operate at low current densities (< 5 mA cm^−2^) and capacities (< 5 mAh cm^−2^),^[^ ^12^ ^]^ revealing limited lifespan due to inadequate Li^+^ diffusion kinetics.^[^ ^13^ ^]^ To build high‐rate batteries, we need to design a 3D host with enhanced Li^+^ diffusion efficiency. Certain Li_x_M alloys, including Li_13_Sn_5_, LiZn, and Li_13_In_3_, etc. have shown promise with superior Li wettability and amplified Li^+^ diffusion kinetics, all without undergoing intercalation chemistry.^[^ ^14^ ^]^ Among them, Li_x_Sn_y_ alloy has higher decomposition potential ≈0.3 V and less side reactions with the electrolyte, making it a viable scaffold for Li deposition.^[^ ^10^, ^15^ ^]^ For example, Li_22_Sn_5_ and Li_5_Sn_2_ have been successfully transferred to the 3D frameworks (e.g., Ni foam, carbon fiber, etc.) as host for Li deposition, which relieves the dendrite issue upon cycling.^[^ ^16^ ^]^ Despite the prolonged cycle performance, the existing 3D Li_x_Sn_y_ alloy scaffolds still suffer from architectural flaws that compromise scaffold stability, low DOD, and lead to excessive Li consumption during pre‐lithiation — challenges that need addressing.

In this work, we develop a conformal 3D flexible Li_13_Sn_5_ scaffold for dendrite‐free and high DOD Li plating/stripping. By the employment of SnO_2_ nanocrystals uniformly distributed on CC, the electrode achieves a low SnO_2_ content (only 2 wt.%) which stands out among previously reported Sn‐based anodes (>5 wt.%), thus achieving a significantly minimized lithium consumption during prelithiation (only 0.6 wt.%). Experimental results indicate that the conformal 3D Li_13_Sn_5_ framework guarantees fast electron transfer and ion diffusion. Owing to these enhancements, dendrite growth is substantially mitigated, as corroborated by in situ optical microscopy, ex situ structural analyses, and multi‐physics field simulations. As a result, the Li/Li_13_Sn_5_@CC symmetric cells exhibit outstanding performance (enduring 5 mA cm^−2^ and 5 mAh cm^−2^ for 2600 h) and a remarkable DOD of 87.1% (lasting 40 mAh cm^−2^ for 600 h). In a practical context, our flexible Li/Li_13_Sn_5_@CC||Li_2_S_6_/SnO_2_@CC pouch cell delivers an areal capacity of 5.04 mAh cm^−2^ and an impressive areal energy density of 10.6 mWh cm^−2^, even powering electric fans under various deformation states. This work paves a new venue for crafting flexible LMAs optimized for both high‐rate capability and superior DOD.

## 2 Results and Discussion

**Figure 1:**
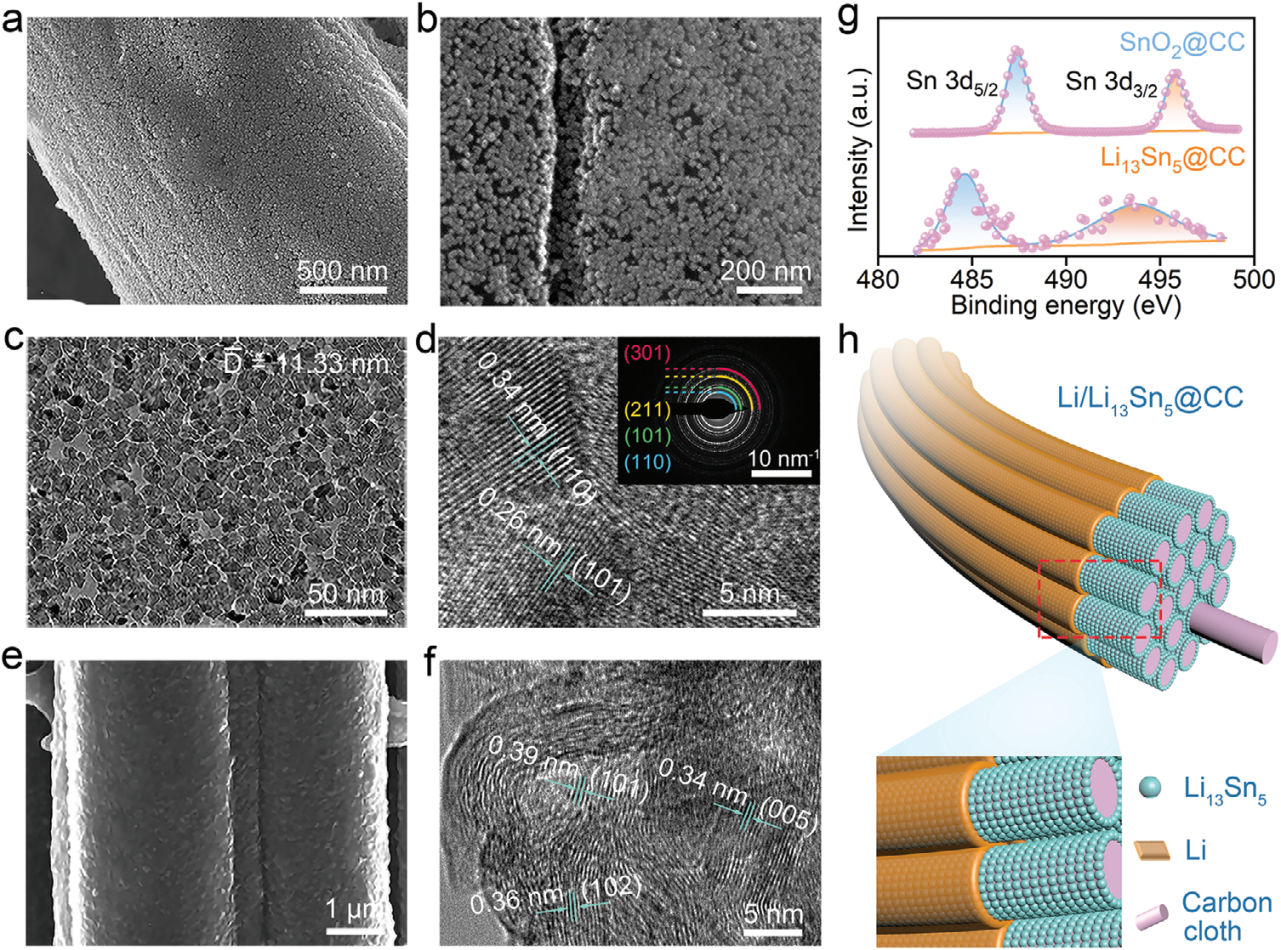
a) SEM and b) zoomed‐in images of SnO_2_@CC. c) TEM and d) HRTEM images of SnO_2_ distributed on CC. Inset is the corresponding SAED pattern. e) SEM and f) HRTEM images of Li_13_Sn_5_@CC. g) High‐resolution XPS spectra of Sn 3d for SnO_2_@CC and Li_13_Sn_5_@CC. h) Schematic illustration of Li_13_Sn_5_@CC and the associated Li deposition.

SnO_2_@CC was synthesized through a straightforward hydrothermal method, followed by an annealing process.^[^ ^17^ ^]^ Subsequently, the lithium source was incorporated via molten Li infusion at 350 °C. In the initial step, scaffolds with varying SnO_2_ loadings were produced by modulating the precursor solution's concentration. Scanning electron microscopy (SEM) images vividly display the morphology of pristine CC (Figure S1a, Supporting Information), SnO_2_ nanocrystals on CC (SnO_2_@CC, **Figure** **1**a), rodlike SnO_2_ on CC (R‐SnO_2_@CC, Figure S1b, Supporting Information) and flower‐like SnO_2_ on CC (F‐SnO_2_@CC, Figure S1c, Supporting Information).^[^ ^18^ ^]^ The morphological distinctions among these structures can be attributed to the variation in SnO_2_ content. Accordingly, the content of SnO_2_ in pristine CC, SnO_2_@CC, R‐SnO_2_@CC, and F‐SnO_2_@CC is calculated to be 0, 2, 8.3, and 14 wt.%, respectively, as confirmed by thermogravimetric analysis (TGA, Figure S2a, Supporting Information).^[^ ^19^ ^]^ X‐ray diffraction (XRD, Figure S2b, Supporting Information) results further confirm the identified phases, aligning perfectly with rutile SnO_2_ (JCPDS No. 41–1445). The diffraction peaks of SnO_2_@CC exhibit reduced intensity when compared with R‐SnO_2_@CC and F‐SnO_2_@CC, which is a consequence of its diminished SnO_2_ content.^[^ ^20^ ^]^ The strategy of reducing SnO_2_ content has proven efficient in curtailing both pre‐lithiation capacity and the scaffold's irreversible capacity. To evaluate the latent capacity retained by these scaffolds, they were initially plated to a capacity of 6 mAh cm^−2^ at a rate of 0.5 mA cm^−2^ and subsequently stripped down to a 1 V cut‐off (Figure S3, Supporting Information). This process reveals a descending trend in both pre‐lithiation (from 6 to 2.7 to 2.3 to 2.1 mAh cm^−2^) and irreversible capacities (from 2.1 to 1.5 to 0.3 to 0.23 mAh cm^−2^) in sync with the diminishing SnO_2_ content (Table S1, Supporting Information). Importantly, the SnO_2_@CC scaffold outperforms both the R‐SnO_2_@CC, F‐SnO_2_@CC, and pristine CC in terms of Coulombic efficiency and long‐term capacity retention, showcasing its structural robustness (Figure S4, Supporting Information). In essence, the SnO_2_@CC scaffold, with its remarkably low SnO_2_ loading of 2 wt.%, successfully minimizes the pre‐lithiation and irreversible capacities, thereby reserving a more substantial capacity for subsequent cycles. Based on these findings, SnO_2_@CC was chosen as the composite Li anode's scaffold for in‐depth analysis of Li deposition behavior and electrochemical attributes.

The SnO_2_ nanocrystals on CC display a notably dense and consistent distribution, as clearly depicted in the zoomed‐in SEM image (Figure 1b). Furthermore, the transmission electron microscopy (TEM) image (Figure 1c), combined with the particle size distribution histogram (Figure S5, Supporting Information), unveils nanoparticles with an impressively fine average diameter of 11.33 nm, significantly enhancing the exposure of the active specific surface area.^[^ ^21^ ^]^ The lattice spacings shown in the high‐resolution TEM (HRTEM) image (Figure 1d), along with the selected area electron diffraction (SAED) pattern (Inset Figure 1d), resonate perfectly with the (110) and (101) crystal facets of rutile SnO_2_. Such a surface feature facilitates the rapid infusion of molten Li into the scaffold in a mere 20 s (Video S1, Supporting Information). In stark contrast, pristine CC resists molten Li penetration, even when subjected to deliberate pressure and friction (Video S2, Supporting Information). Significantly, this is a pioneering instance where a scaffold with such minuscule SnO_2_ content has successfully facilitated molten Li infusion, thanks to the expansive specific surface area of the ultrafine SnO_2_ nanoparticles. Following the molten Li's infusion, the composite Li electrode's surface adopts a silvery sheen and showcases a uniform metallic Li coating (Figure S6a,b, Supporting Information). This electrode is also noteworthy for its exceptional flexibility, demonstrated by its resilience to twisting and bending (Figure S6c, Supporting Information). When fully stripped to 1 V, the capacity–voltage curve of the composite Li electrode reveals a total areal capacity of 45.9 mAh cm^−2^ (Figure S6d, Supporting Information). When Li stripping to 0.1 V (Figure S6e, Supporting Information), a uniform layer is observed to be evenly spread on the CC (Figure 1e), which is identified as Li_13_Sn_5_, as corroborated by HRTEM (Figure 1f). The lattice spacings, specifically d_(101)_ = 0.39 nm, d_(102)_ = 0.36 nm, and d_(005)_ = 0.34 nm, align seamlessly with the crystal facets characteristic of Li_13_Sn_5_. In addition, the high‐resolution X‐ray photoelectron spectroscopy (XPS) spectrum of Sn 3d (Figure 1g) spotlight a shift in the characteristic peaks of Sn 3d_5/2_ and Sn 3d_3/2_ from 487.3 and 495.7 eV in SnO_2_@CC, respectively, to 484.2 and 492.6 eV in Li_13_Sn_5_@CC. These findings match well with the TEM observations. Additionally, after the reactions, the uniformity of Sn element on CC remains unchanged, as evidenced by SEM and elemental mapping analyses (Figure S7, Supporting Information). In this context, the conformal coating of Li_13_Sn_5_ promotes preferred Li nucleation and ensures uniform Li deposition within the scaffolds (Figure 1h).

**Figure 2:**
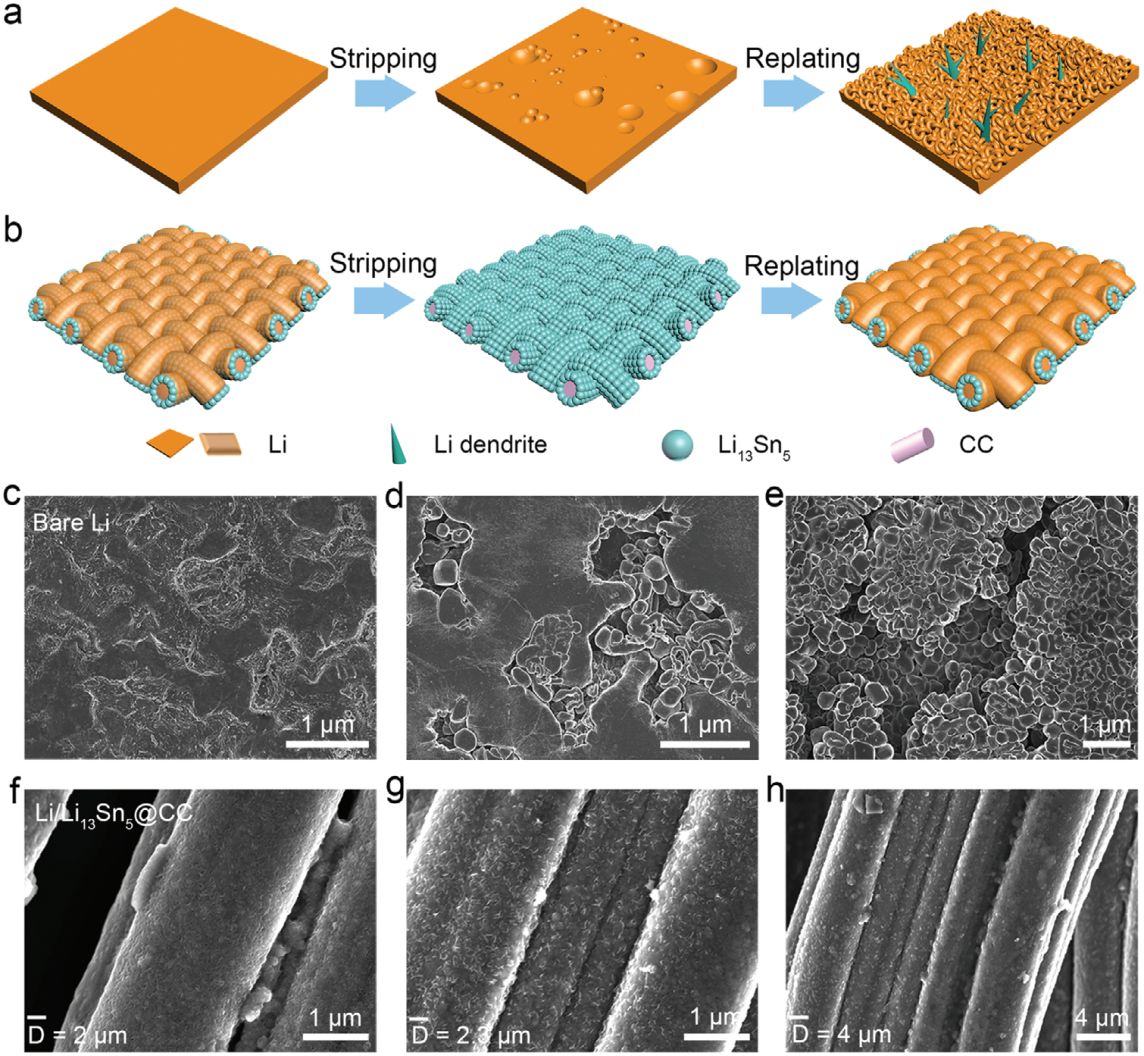
Schematic illustration of the Li stripping and replating behavior on a) bare Li and b) Li/Li_13_Sn_5_ electrodes. c) SEM image of the stripped bare Li. SEM images of replating d) 5 and e) 10 mAh cm^−2^ Li on the stripped bare Li. f) SEM image of the stripped Li/Li_13_Sn_5_@CC. SEM images of replating g) 5 and h) 10 mAh cm^−2^ Li on the Li/Li_13_Sn_5_@CC.

As schematically shown in **Figure** **2**a,b, the bare Li electrode displays irregular stripping, detrimental dendrite growth, and significant lithium volume expansion. These issues originate from the uneven lithium ion flux and the inherent host‐less characteristic of lithium. Conversely, a uniform Li_13_Sn_5_ layer acts as a barrier against dendrite formation and substantial Li volume changes, ensuring a consistent Li^+^ flux and promoting uniform Li stripping/plating. Subsequently, the Li deposition patterns on stripped bare Li and Li/Li_13_Sn_5_@CC were analyzed using SEM.^[^ ^22^ ^]^ As evident in Figure 2c,d, numerous pits form on bare Li during the Li stripping process, and Li tends to redeposit preferentially on these pits during plating. Over time, this leads to the formation of extensive Li dendrites (Figure 2e). Intriguingly, the Li/Li_13_Sn_5_@CC retains a consistent Li_13_Sn_5_ layer even after complete Li stripping, as illustrated in Figure 2f. Owing to the guiding properties of the conformal Li_13_Sn_5_ layer, the surface of the electrode maintains a uniform Li deposition, as depicted in Figure 2g,h.

To assess the wettability of the electrolyte on the electrodes, contact angles were measured by vertically dripping an ether‐based electrolyte onto both bare Li and Li/Li_13_Sn_5_@CC. The observed contact angles were 30° for the bare Li and ≈0° for the Li/Li_13_Sn_5_@CC (Figure S8, Supporting Information), which suggests that Li_13_Sn_5_ layer is beneficial to rapid electrolyte penetration and the electrolyte can fully permeate the Li/Li_13_Sn_5_@CC electrode.^[^ ^23^ ^]^ The wetting free energy^[^ ^1^ ^]^ of the two electrodes was then calculated, which indicates that the Li/Li_13_Sn_5_@CC anode delivers a higher ∆G value of 1658.64 J mol^−1^, which exceeds the value of bare Li (787.8 J mol^−1^) (Figure S9, Supporting Information). Additionally, both the bare Li and Li/Li_13_Sn_5_@CC electrodes were immersed in the electrolyte for 1 min to examine the elemental distribution within the electrodes. As shown in Figure S10 (Supporting Information), the F and S elements were only present on both sides of the bare Li, indicating that the electrolyte doesn't penetrate the inner bare Li electrode. By contrast, the Sn, C, F, and S elements were uniformly distributed throughout the Li/Li_13_Sn_5_@CC electrode (Figure S11, Supporting Information), highlighting the electrode's superior electrolyte wettability. This result suggests that metallic Li can directly dissolve from and plate onto the Li_13_Sn_5_@CC scaffold. The transference numbers (t) were further accurately calculated by the Bruce‐Vincent method to evaluate the Li^+^ diffusion ability (Figure S12, Supporting Information).^[^ ^24^ ^]^ Notably, the Li/Li_13_Sn_5_@CC symmetric cell provides a high Li^+^ transference number of 0.76, strongly confirming the excellent Li diffusion kinetics. In contrast, the t value of the bare Li is as low as 0.24, which is due to the unrestricted migration of anions. The Li^+^ diffusion process can be verified by chronoamperometric (CA) curves (Figure S13, Supporting Information). At an overpotential of −150 mV, the current density of bare Li continues to increase over time, indicating that the deposition diffusion process is a long planar diffusion process. This diffusion pattern leads to severe dendrite growth during charging and discharging due to the “tip effect”. In sharp contrast, Li/Li_13_Sn_5_@CC only experienced a planar diffusion and nucleation process for 2 s before achieving a stable current density, indicating that the main diffusion method is mainly 3D diffusion. The CA results show that the Li_13_Sn_5_ layer indeed facilitates uniform diffusion of Li^+^, thereby achieving uniform Li deposition and excellent electrochemical performance.

**Figure 3:**
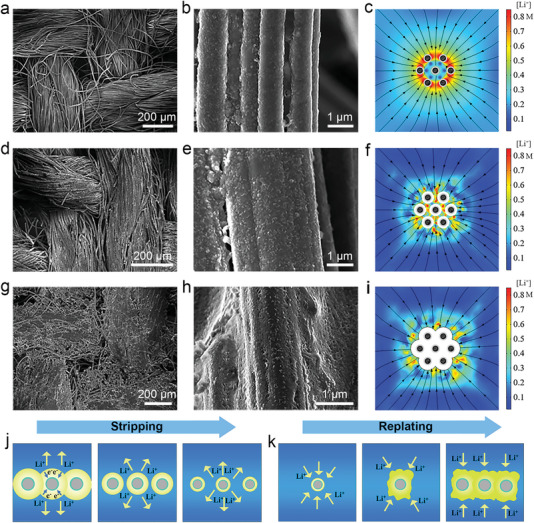
a,b) SEM images and c) COMSOL simulation of the Li/Li_13_Sn_5_@CC electrode after Li‐fully stripping. d,e) SEM images and f) COMSOL simulation of replating 5 mAh cm^−2^ Li on the Li_13_Sn_5_@CC electrode. g,h) SEM images and i) COMSOL simulation of replating 20 mAh cm^−2^ Li on the Li_13_Sn_5_@CC electrode. Schematic illustration of the j) Li stripping and k) replating processes of the Li_13_Sn_5_@CC electrode.

To delve into the Li deposition behavior on 3D Li_13_Sn_5_@CC scaffold at the electrode level, we further employed both SEM observation and multi‐physics field simulations. The SEM images at both low and high magnifications reveal a smooth Li_13_Sn_5_ layer covering the CC (**Figure** **3**a,b), evident after complete Li stripping. The COMSOL simulations indicate that this flat surface promotes a uniform electric field. While the Li_13_Sn_5_ layer results in a heightened Li^+^ concentration near the CC's surface, thereby enhancing Li^+^ diffusion kinetics (Figure 3c). Hence, Li_13_Sn_5_ alloy on CC, which can act as seeds for homogeneous Li nucleation and growth, thereby enabling Li^+^ anchored in the scaffold. Upon replating 5 mAh cm^−2^ Li, the SEM images show that Li deposits conformally on the scaffold, with a noticeable increase in diameter (Figure 3d,e). This uniform Li deposition pattern aligns with the COMSOL findings (Figure 3f).^[^ ^25^ ^]^ When the replating capacity is increased to 20 mAh cm^−2^, the corresponding SEM images highlight a dendrite‐free Li deposition morphology (Figure 3g,h). This is attributed to the consistent electric field distribution, a result of the uniform Li deposition in the early stages, which ultimately fosters optimal Li deposition in subsequent stages. The COMSOL simulations state that the Li_13_Sn_5_@CC, serving as an electric transfer path, maintains a consistent electric field even after Li is uniformly restored on the electrode (Figure 3i). Figure 3j,k provides a schematic representation of the Li plating and stripping processes. Thanks to the intertwined and distinct electric transfer paths, coupled with the uniform electrolyte/electrode interface of Li/Li_13_Sn_5_@CC, metallic Li remains confined within the 3D scaffold, ensuring a dendrite‐free Li deposition behavior. We further provide the cross‐section SEM image of Li/Li_13_Sn_5_@CC electrode operating at a high DOD of 87.1% (40 mAh cm^−2^). As shown in Figure S14 (Supporting Information), when stripped for 40 mAh cm^−2^, it can be seen that the Li/Li_13_Sn_5_@CC presents a uniform cross‐sectional morphology. Then when it is redeposited for 40 mAh cm^−2^, the morphology manifests uniform and dense lithium deposition without dendrite growth.

**Figure 4:**
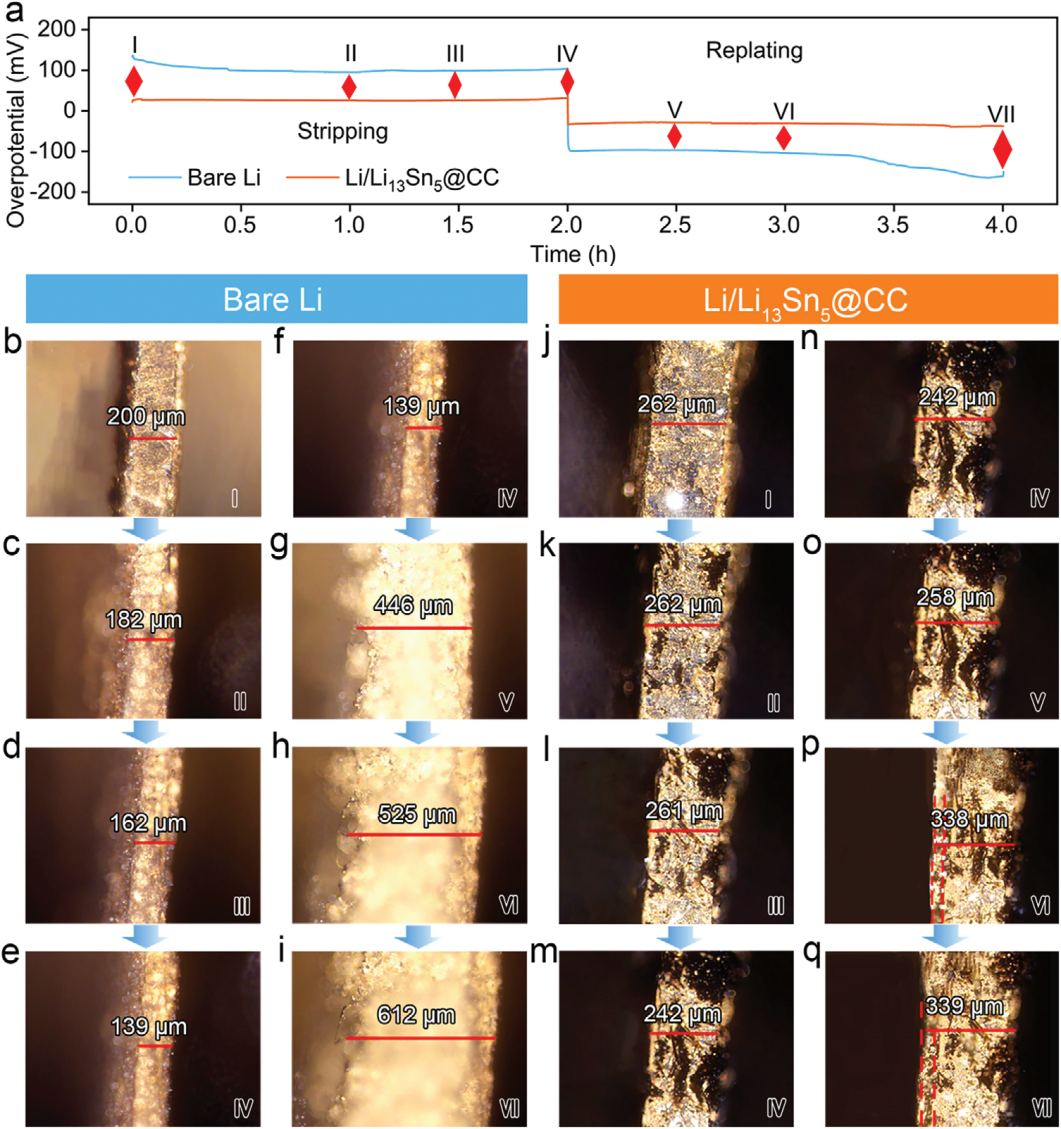
In situ optical microscopy analyses of the Li stripping and replating processes in the electrodes. a) The galvanostatic charge/discharge voltage profiles of bare Li and Li/Li_13_Sn_5_@CC electrode at 5 mA cm^−2^ with a fixed capacity of 10 mAh cm^−2^. The photographs of bare Li at different b–e) stripping and f–i) replating states. The photographs of Li/Li_13_Sn_5_@CC at different j–m) stripping and n–q) replating states.

In situ optical microscopy was utilized to provide a clear visualization of the Li stripping/plating processes in these electrodes.^[^ ^26^ ^]^ Both Li/Li_13_Sn_5_@CC and bare Li were set up vertically as symmetric cells within a sealed apparatus, undergoing a 2 h stripping at 5 mA cm^−2^, followed by a 2 h replating process. The cross‐sectional images showcasing the electrode stripping/plating states (I–VII) align with the galvanostatic charge/discharge voltage profile depicted in **Figure** **4**a. As illustrated in Figure 4b–e, throughout the Li stripping phase, Li dissolved from both sides of the bare Li electrode, leaving a non‐uniform surface due to inconsistent electric/ionic flux. During the replating phase (Figure 4f–i), Li tended to deposit preferentially at hot spot sites, further exacerbating surface irregularities. This cycle underscores the challenges inherent to the Li host‐less nature.^[^ ^10^ ^]^ Conversely, as highlighted by the red annotations in Figure 4j–m, a portion of Li steadily dissolved from the entirety of the Li/Li_13_Sn_5_@CC electrode, including its interior. This behavior stems from the superior electrolyte wettability of the Li/Li_13_Sn_5_@CC electrode. Accordingly, the conductive 3D scaffold, boasting a large specific area, effectively redistributes the electric/ionic flux and reduces local current density. In addition, the uniform Li_13_Sn_5_ layer not only facilitates faster Li^+^ diffusion kinetics but also directs consistent Li stripping and plating, achieving dendrite‐free Li deposition within the 3D architecture. Given that Li tends to replat in areas with rapid electron transfer and elevated binding energy, it remains well‐contained within the host, as shown in Figure 4n–q. This disrupts the previously mentioned detrimental cycle. As anticipated, the Li/Li_13_Sn_5_@CC electrode effectively curbs the unchecked volume expansion of Li. Figure 4i,q displays the cross‐sectional fluctuations of the bare Li and Li/Li_13_Sn_5_@CC after 10 mAh cm^−2^ Li replating, as observed in situ. The bare Li presents a rough and loose morphology with a significant dimensional change, whereas the Li/Li_13_Sn_5_@CC shows much less volume fluctuation. This favorable outcome is attributed to the Li_13_Sn_5_ layer, which enhances the rapid mass/charge transfer of the scaffold. Additionally, the intertwined CC offers ample space to contain the Li within the 3D host, collaboratively leading to minimal volume variation.

**Figure 5:**
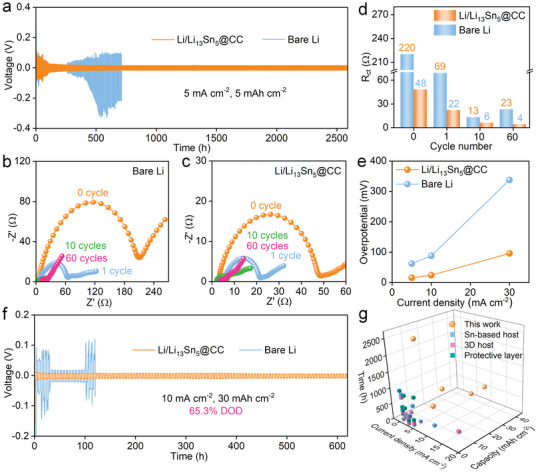
Electrochemical performance of bare Li and Li/Li_13_Sn_5_@CC in symmetric cells. a) Cycling performance at 5 mA cm^−2^ for 5 mAh cm^−2^. Nyquist plots of b) bare Li and c) Li/Li_13_Sn_5_@CC. d) *R*_ct_ of the symmetric cells after different cycles. e) Overpotential of the cells at different current density for 5 mAh cm^−2^. f) Cycling performance at 10 mA cm^−2^ for 30 mAh cm^−2^. g) Comparison of Li/Li_13_Sn_5_@CC with recently reported Sn‐based LMAs.

To assess the Li/Li_13_Sn_5_@CC electrode's suitability for high‐power‐density applications, galvanostatic Li plating/stripping tests were conducted at escalating current densities of 5, 10, and 30 mA cm^−2^, using a practical application capacity of 5 mAh cm^−2^.^[^ ^27^ ^]^ The electrochemical performances were evaluated in symmetric cells equipped with two identical electrodes. When cycling at 5 mA cm^−2^ (**Figure** **5**a), a noticeable smooth voltage drop appears in the early stages, attributed to the activation of the passivation layer and the formation of a solid electrolyte interphase (SEI). Notably, the Li/Li_13_Sn_5_@CC electrode maintains a consistent overpotential of ≈15.8 mV for 2600 h. In contrast, the Li anode's overpotential exhibits significant fluctuations after 200 h, leading to cell failure. In addition, we also provided the enlarged voltage–time curves in Li/Li_13_Sn_5_@CC symmetric cells after long cycles. As shown in Figure S15 (Supporting Information), the Li/Li_13_Sn_5_@CC electrode exhibits a small and steady overpotential of ≈18 mV even after 2000 h. For a closer look at the electrode interface, Nyquist plots were generated after 0, 1, 10, and 60 cycles (Figure 5b,c), with the *R*_ct_ variation trend for symmetric cells illustrated in Figure 5d. The plot reveals a decreasing *R*_ct_ trend for the Li/Li_13_Sn_5_@CC electrode, in contrast to the bare Li's initial decline followed by an increase, aligning with the voltage profile. These findings suggest that the Li/Li_13_Sn_5_@CC electrode possesses a stable SEI with minimal interfacial impedance, indicative of dendrite‐free Li deposition without dead Li accumulation.^[^ ^28^ ^]^ When subjected to a higher current density of 10 mA cm^−2^ for 5 mAh cm^−2^ (Figure S16a, Supporting Information), the Li/Li_13_Sn_5_@CC electrode showcases a stable overpotential of ≈24 mV for 800 h. Conversely, the bare Li anode could not withstand this elevated current density, displaying significant voltage fluctuations after just 30 h and subsequent cell failure. Unlike bare Li, which restricts Li stripping/plating to its surface, the Li/Li_13_Sn_5_@CC facilitates this process along numerous internal mass/charge transfer paths, addressing the mismatch between electrode electrons and nearby SEI Li ions. Further tests at an ultra‐high current density of 30 mA cm^−2^ (Figure S16b, Supporting Information) reveal that the Li/Li_13_Sn_5_@CC electrode delivers exceptional electrochemical performance, maintaining a stable voltage hysteresis of ≈192 mV over 40 h with negligible voltage variations. Post‐mortem SEM analysis after 50 cycles indicates that the bare Li disintegrated, with extensive filamentous Li dendrite growth on its surface (Figure S17a, Supporting Information). On the contrary, the Li/Li_13_Sn_5_@CC electrode displays a smooth surface devoid of Li accumulation (Figure S17b, Supporting Information). Additionally, the overpotential of the Li/Li_13_Sn_5_@CC symmetric cells rises only slightly with increased current density, significantly surpassing its counterpart (Figure 5e). To further investigate the condition of dead and circulating electrodes after extensive cycling, SEM was utilized to analyze the morphology of the bare Li electrode upon failure and the Li/Li_13_Sn_5_@CC electrode after 2600 h of cycling at 5 mA cm^−2^ and 5 mAh cm^−2^. Figure S18 (Supporting Information) vividly illustrates the contrast: the Li/Li_13_Sn_5_@CC anode exhibits uniform and dense lithium deposition after long‐term cycling (≈2600 h), whereas the surface of the bare Li anode becomes markedly uneven with significant dendritic growth after only 700 h of cycling. This clearly demonstrates that the continual dendrite growth rapidly compromises the symmetrical batteries with bare Li, while the Li_13_Sn_5_‐induced uniform lithium deposition extends battery life. To evaluate the stability of the bare Li and Li/Li_13_Sn_5_@CC anodes, rate performance tests were conducted.^[^ ^29^ ^]^ As shown in Figure S19 (Supporting Information), the symmetrical cell's rate performance at current densities ranging from 1 to 40 mA cm^−2^ was analyzed. The Li/Li_13_Sn_5_@CC battery displays exceptional electrochemical performance, maintaining a stable and low overpotential of just 150 mV even at the high current density of 40 mA cm^−2^. In stark contrast, the bare Li anode quickly failed at even minimal current densities, a failure attributed to the growth of dendrites that pierce the separator. Anode stability was further assessed by cycling bare||Cu and Li/Li_13_Sn_5_||Cu half cells at 5 mA cm^−2^ for 5 mAh cm^−2^. As indicated in Figure S20 (Supporting Information), the Li/Li_13_Sn_5_||Cu half‐cell exhibits an impressive initial Coulombic efficiency of 95%, which rapidly increases to a stable Coulombic efficiency of 99.5% over 120 cycles. Conversely, the bare Li||Cu half‐cell failed after just 50 cycles, primarily due to extensive dendritic growth.

The DOD of the electrode directly influences the energy density of a battery. However, a greater DOD amplifies volume expansion, accelerating cell failure.^[^ ^30^ ^]^ To further assess the Li/Li_13_Sn_5_@CC electrode's viability for high‐energy‐density applications, symmetric cells were tested at a current density of 5 mA cm^−2^ with DOD set at 43.6% (20 mAh cm^−2^), and a high current density of 10 mA cm^−2^ with DOD set at 65.3% (30 mAh cm^−2^) and 87.1% (40 mAh cm^−2^). When cycling at 5 mA cm^−2^ for 20 mAh cm^−2^ (Figure S21a, Supporting Information), obviously, the Li/Li_13_Sn_5_@CC electrode maintains a consistent overpotential of ≈10.5 mV for 800 h. In contrast, the bare Li anode's overpotential exhibits significant fluctuations after 400 h, leading to cell failure. When cycling at a substantial capacity of 30 mAh cm^−2^ (Figure 5f), the bare Li anode quickly deteriorates due to dead Li accumulation and electrode fragmentation caused by significant volume expansion. In stark contrast, the Li/Li_13_Sn_5_@CC electrode showcases prolonged stable cycling, maintaining an overpotential of 9.6 mV for 600 h. The capacity of the Li/Li_13_Sn_5_@CC symmetric cell was further increased to 40 mAh cm^−2^ (an unprecedented DOD of 87.1%) at a current density of 10 mA cm^−2^ (Figure S21b, Supporting Information). The voltage profile displays a minimal overpotential of 18 mV, sustaining stable cycling for 600 h. Figure 5g compares the Li/Li_13_Sn_5_@CC electrode with other recently reported Li anodes, whether they were developed using Sn‐based hosts,^[^ ^17^, ^31^ ^]^ designed as 3D hosts,^[^ ^19^, ^23^, ^28^, ^32^ ^]^ or modified with protective layers.^[^ ^18^, ^25^, ^32^ ^]^ Clearly, the Li/Li_13_Sn_5_@CC electrode's electrochemical performance is among the best, as summarized in Table S2 (Supporting Information). The exceptional performance of the Li/Li_13_Sn_5_@CC electrode can be attributed to the Li_13_Sn_5_ layer, which promotes dendrite‐free Li deposition and enhances Li^+^ diffusion kinetics at the SEI. Besides, the interwoven carbon fibers offer ample space to comfortably accommodate electrode volume changes.

**Figure 6:**
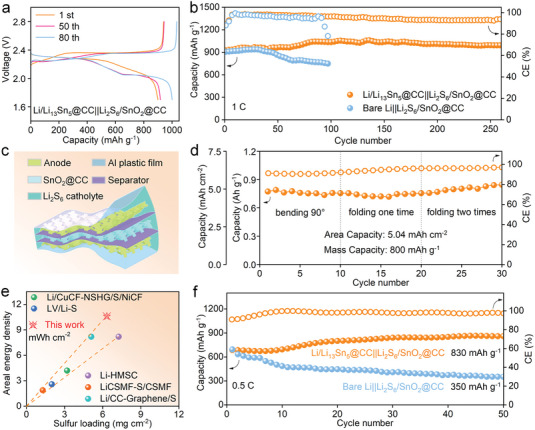
a) Charge–discharge voltage curves of Li/Li_13_Sn_5_@CC||Li_2_S_6_/SnO_2_@CC cell at different cycles. b) Cycling performance of Li/Li_13_Sn_5_@CC||Li_2_S_6_/SnO_2_@CC and bare Li||Li_2_S_6_/SnO_2_@CC cells at 1 C. c) Schematic illustration of the layered structure of the flexible Li/Li_13_Sn_5_@CC||Li_2_S_6_/SnO_2_@CC pouch cell. d) Li/Li_13_Sn_5_@CC||Li_2_S_6_/SnO_2_@CC pouch cell tested at different deformation states of 0.1 C. e) The profile of energy density and sulfur loading comparison of Li/Li_13_Sn_5_@CC||Li_2_S_6_/SnO_2_@CC pouch cell with recently reported ones. f) Cycling performance of Li/Li_13_Sn_5_@CC||Li_2_S_6_/SnO_2_@CC and bare Li||Li_2_S_6_/SnO_2_@CC pouch cells at 0.5 C.

Li/Li_13_Sn_5_@CC anodes were paired with SnO_2_@CC current collectors in Li_2_S_6_ catholyte to realize their potential in high‐energy‐density flexible Li||Li_2_S_6_/SnO_2_@CC full cells. The sulfurophilic SnO_2_@CC, due to the polysulfide chemisorption of SnO_2_, effectively confines S within the 3D scaffold.^[^ ^33^ ^]^ The combinations of SnO_2_@CC with Li_2_S_6_ catholyte paired with Li/Li_13_Sn_5_@CC and bare Li anodes are referred to as Li/Li_13_Sn_5_@CC||Li_2_S_6_/SnO_2_@CC and bare Li||Li_2_S_6_/SnO_2_@CC, respectively. The discharge plateaus observed at 2.3 and 2.1 V align well with the Li/Li_13_Sn_5_@CC||Li_2_S_6_/SnO_2_@CC cell (**Figure** **6**a). Notably, the specific capacity of Li/Li_13_Sn_5_@CC||Li_2_S_6_/SnO_2_@CC full cells exhibits an upward trend, attributed to the catholyte's activation process. This is further evidenced by the pronounced improvement in the second plateau capacity at 2.1 V by the 80th cycle. In sharp contrast, the bare Li|| Li_2_S_6_/SnO_2_@CC cell displays a consistent capacity decline (Figure S22, Supporting Information), probably stemming from the ongoing creation of disconnected dead Li and S. Additionally, the reduced voltage hysteresis of Li/Li_13_Sn_5_@CC||Li_2_S_6_/SnO_2_@CC, as indicated by the narrower voltage gap between the charge/discharge curve, suggests faster Li^+^ transfer near the electrode/electrolyte interface (Figure S23, Supporting Information).

The long‐term cycling performance of the Li/Li_13_Sn_5_@CC||Li_2_S_6_/SnO_2_@CC coin cell was exceptional, maintaining 1050 mAh cm^−2^ over 250 cycles at 1 C (Figure 6b). In comparison, the bare Li||Li_2_S_6_/SnO_2_@CC experiences rapid capacity decay after just 50 cycles, with abrupt failure by the 98th cycle. The rate performance of the full cells was also studied (Figure S24, Supporting Information). At a current density of 3 C, the capacity of Li/Li_13_Sn_5_@CC||Li_2_S_6_/SnO_2_@CC is ≈750 mA h g^−1^, which is higher than that of bare Li||Li_2_S_6_/SnO_2_@CC battery (680 mA h g^−1^). More importantly, when the current density returns to 0.2 C, the specific capacity of the Li/Li_13_Sn_5_@CC||Li_2_S_6_/SnO_2_@CC battery stabilizes at 980 mA h g^−1^, while the capacity of the bare Li||Li_2_S_6_/SnO_2_@CC battery decreases.

Deformability and power endurance are two key factors for evaluating the performances of flexible batteries.^[^ ^34^ ^]^ Accordingly, a flexible Li/Li_13_Sn_5_@CC||Li_2_S_6_/SnO_2_@CC pouch cell was conceptualized, as depicted in Figure 6c. This pouch cell was then evaluated under various deformation states, such as 90° bending, onefold, and twofold (Figure 6d; Figure S25, Supporting Information). Impressively, the cell's capacity shows slightly changes as deformation lessened, showcasing remarkable deformability. Besides, the pouch cell could power electric fans under all deformation states (Video S3, Supporting Information), highlighting its potential for power batteries. Moreover, the pouch cell's energy density (10.6 mWh cm^−2^) ranks prominently among recent studies (Figure 6e; Table S3, Supporting Information).^[^ ^33^, ^34^, ^35^ ^]^ The cycling performance of the pouch cell is presented in Figure 6f, which maintains a capacity of 830 mAh g^−1^ after 50 cycles. This result is significantly better than that of the bare Li||Li_2_S_6_/SnO_2_@CC cell, which only retains 350 mAh g^−1^ at the same cycle count. This favorable outcome is attributed to the anode's dendrite‐free nature and controlled dimensional fluctuation.^[^ ^36^ ^]^ Additionally, these anodes were paired with the commercial LiFePO_4_ (LFP) cathode. As illustrated in Figure S17 (Supporting Information), the Li/Li_13_Sn_5_@CC||LFP cell showcases a high capacity of 120 mAh g^−1^ over 500 cycles and superior rate performance with reduced voltage hysteresis. The unique structure and properties of Li/Li_13_Sn_5_@CC electrode address the key challenges of LMAs, offering great potential to fabricate high‐energy‐density batteries.

## 3 Conclusion

In summary, we have introduced a 3D flexible Li_13_Sn_5_ scaffold as an exceptional dendrite inhibitor, paving the way for high‐rate and high DOD electrodes. With a minimal SnO_2_ content (2 wt.% in the scaffold), there is only a trace amount of Li consumed during pre‐lithiation. Both experimental data and COMSOL simulations reveal that the Li_13_Sn_5_ scaffold facilitates rapid and uniform Li deposition, thanks to enhanced Li^+^ diffusion kinetics and consistent electronic field distribution. As a result, the symmetric cell utilizing the Li/Li_13_Sn_5_@CC electrode demonstrates a remarkable lifespan of 2600 h without voltage fluctuations, even at a high rate of 5 mA cm^−2^ for 5 mAh cm^−2^. Impressively, the electrode attains an ultra‐high DOD of 87.1% (40 mAh cm^−2^ at 10 mA cm^−2^), maintaining its performance for 600 h. Furthermore, when integrated into a Li/Li_13_Sn_5_@CC||Li_2_S_6_/SnO_2_@CC pouch cell, the electrode delivers an outstanding areal energy density of 10.6 mWh cm^−2^, maintaining this even after 30 cycles under challenging deformation conditions. This research illuminates a novel approach to achieving high areal energy/power density anodes by enhancing utilization efficiency and providing consistent lithiophilic guidance.

## 4 Experimental Section

### Preparation of SnO_2_@CC

SnO_2_@CC was fabricated utilizing a straightforward hydrothermal and annealing procedure. Initially, a pristine CC was submerged in piranha solution for 2 h. Subsequently, tin chloride dihydrate (0.0025 mmol) and sodium citrate (0.05 mmol) were dissolved in 10 mL of deionized water. Following this, a sodium hydroxide solution (0.004 m, 5 mL) was gradually added to the mixture, ensuring thorough stirring of the precursor solution. Both the precursor solution and the treated CC were then placed into a Teflon‐lined stainless steel autoclave and maintained at 180 °C for 12 h. Afterward, the product was washed with deionized water and subjected to ultrasonication for 1 min to detach any loosely adhered SnO_2_ from the CC. Finally, the products underwent calcination at 300 °C for 4 h with a heating rate of 2 °C min^−1^.

### Preparation of R‐SnO_2_@CC and F‐SnO_2_@CC

F‐SnO_2_@CC was synthesized following the same procedure, with the exception of using a precursor solution with five times and tenfold concentration for the hydrothermal process.

### Preparation of Li/Li_13_Sn_5_@CC and Li_13_Sn_5_@CC

Li/Li_13_Sn_5_@CC electrodes were fabricated by infusing molten Li into the SnO_2_@CC scaffold within a glove box, maintaining H_2_O and O_2_ content <0.1 ppm. Initially, 15 mg of Li was melted at 300 °C. Subsequently, a disc of SnO_2_@CC, measuring 12 mm in diameter, was placed over the molten Li. Following the infusion of molten Li, the newly prepared Li anode was allowed to cool down, resulting in the formation of Li/Li_13_Sn_5_@CC.

Li_13_Sn_5_@CC was obtained by fully stripping Li/Li_13_Sn_5_@CC to 0.1 V in a coin cell. Subsequently, the electrode was washed with absolute ethanol.

### Assembly of Symmetric and Full Cells

Symmetric cells were assembled in 2032‐type coin cells using two identical Li/Li_13_Sn_5_@CC electrodes within an Ar‐filled glove box, with a control cell assembled under identical conditions using Li foil. A microporous polypropylene film (Celgard, 2400) served as the separator, and the electrolyte was a solution of 1 m LiTFSI in a 1:1 volume mixture of dioxolane (DOL) and dimethoxyethane (DME), containing 1 wt.% LiNO_3_. The Li_2_S_6_ catholyte was prepared by mixing sulfur and Li_2_S powder in a 5:1 mole ratio in a 1:1 volume mixture of DOL and DME. This mixture was stirred at 80 °C for 24 h to form a 4 m dark red Li_2_S_6_ solution, based on the concentration of S. The LFP cathode was fabricated by mixing the active powder, acetylene black, and polyvinylidene difluoride in a mass ratio of 8:1:1 in N‐methyl‐2‐pyrrolidone. This uniform slurry was coated onto Al foil and dried in a vacuum oven at 80 °C overnight, resulting in a mass loading of LFP of ≈2.5 mg cm^−2^.

### Electrochemical Measurements

The galvanostatic tests were executed on a NEWARE battery test system. Electrochemical impedance spectroscopy tests were conducted over frequencies ranging from 0.1 Hz to 100 kHz using a CHI 760 electrochemical workstation. The galvanostatic tests of Li–S and LFP full cells were carried out within a voltage range of 1.7–2.8 and 2.5–4.3 V (vs Li/Li^+^), respectively.

### Materials Characterization

The morphologies of the prepared electrodes were examined using a scanning electron microscope (JEOL JSM‐6700F) and a transmission electron microscope (FEI Talos F200X). The crystalline phase of the electrodes was analyzed using a Bruker D8 Advance diffractometer (Cu Kα radiation). The valence states of the substances were studied by XPS using a ThermoFisher ESCA 250XI (Al Kα radiation). TGA was performed on a Netzsch STA449F3 analyzer under air conditions.

### The Evaluation of Energy Density

Based on the single‐layer pouch cell configuration depicted in Figure 6c, the energy densities *E_g_* ​ and *E_a_* ​ of the single‐layer pouch cell can be calculated using the following equations:

(1) $$E_{g}=\frac{VC}{\sum m_{i}}$$

(2) $$E_{a}=VC$$

Here, *E_g_* ​ and *E_a_* ​ represent the full cell gravimetric and areal energy densities, respectively. *V* denotes the average output voltage, assumed to be 2.1 V, and *C* is the areal capacity (mAh cm^−2^). Each *m_i_*_​_ is the mass per unit area (mg cm^−2^).

### COMSOL Simulation

The distributions of the electric field and Li^+^ concentration were simulated using COMSOL Multiphysics, excluding potential side effects. The entire simulation represents a transient model during the replating process, defined by the mass conservation and electroneutrality conditions of the associated ions (Li^+^ and electrolyte anion). According to the Nernst–Planck equation, the mass conservation equation is represented as:

(3) $$\frac{\partial c_{i}}{\partial t}+\nabla \times N_{i}=0$$

Here, *N_i_* is the flux vector (mol/(m^2^·s)), and *c_i_* is the electrolyte concentration (mol m^−3^). The electroneutrality condition is expressed by the following equation:

(4) $$\sum_{i}z_{i}c_{i}=0$$

Here, *z_i_* represents the ionic charge number. The chemical equivalent coefficients for Li^+^ in the electrolyte and Li atoms on the electrodes were both set to 1.

### Calculation of the Cation Ion Transference Number

Cation transference numbers were determined by integrating alternating‐current (AC) impedance and direct‐current (DC) potentiostatic polarization measurements using symmetric batteries. Initially, the system's state before potentiostatic polarization was designated as the initial state, with the corresponding interface impedance labeled as R_0_. After undergoing prolonged potentiostatic polarization, when the current response trend stabilized, this condition was termed the stable state, characterized by an interface impedance value of R_ss_. AC impedance measurements were promptly conducted as soon as the response current achieved stability, to capture the stable state Nyquist plot. In this plot, the semicircles intersecting the Zre (real impedance) axis provide the corresponding interface impedance values. The cation transference number (𝑡) is calculated using the equation:

(5) $$t=\left(\nabla V/I_{0}-R_{0}\right)/\left(\nabla V/I_{ss}-R_{SS}\right)$$

Here, ΔV represents the applied constant potential (10 mV), I_0_ is the initial response current, I_ss_ denotes the steady‐state response current, and R_0_ and R_ss_ are the electrode interface impedances before and after polarization, respectively.

### Calculation of Li Consumption

As indicated by the equations below, 1 mol SnO_2_ consumes 6.6 mol Li. The amount of SnO_2_ corresponding to 2 wt.% SnO_2_ is multiplied by 6.6, which is the amount of consuming lithium. Then the mass percentage of lithium consumption is converted to 0.6 wt.%.

(6) $$SnO_{2}+4Li^{+}+4e^{-}\to Sn+2Li_{2}O$$

(7) $$5Sn+13Li^{+}+13e^{-}\to Li_{13}Sn_{5}$$

## Conflict of Interest

The authors declare no conflict of interest.

## Supporting information

Supporting Information

Supplemental Video 1

Supplemental Video 2

Supplemental Video 3

## Data Availability

The data that support the findings of this study are available from the corresponding author upon reasonable request.
