# Supplementary material for: Conformal 3D Li/Li13Sn5 Scaffolds Anodes for High‐Areal Energy Density Flexible Lithium Metal Batteries
Source: Adv Sci (Weinh). 2024 Feb 7;11(14):2309254. doi: 10.1002/advs.202309254 (PMC11005696; doi:10.1002/advs.202309254)
Supplement: Supplementary file 1 — Supporting Information [file ADVS-11-2309254-s002.pdf]

ADVANCED SCIENCE
Open Access

Supporting Information

for *Adv. Sci.*, DOI 10.1002/advs.202309254

Conformal 3D Li/$Li_{13}Sn_5$ Scaffolds Anodes for High-Areal Energy Density Flexible Lithium Metal Batteries

*Xiaomei Huo, Xin Gong, Yuhang Liu, Yonghui Yan, Zhuzhu Du and Wei Ai**

Supporting Information

Conformal 3D Li/Li$_{13}$Sn$_5$ Scaffolds Anodes for High-Areal Energy Density Flexible Lithium Metal Batteries

Xiaomei Huo, Xin Gong, Yuhang Liu, Yonghui Yan, Zhuzhu Du and Wei Ai[*]

Frontiers Science Center for Flexible Electronics & Xi'an Institute of Flexible Electronics, Northwestern Polytechnical University, Xi'an 710072, China.

E-mail: iamwai@nwpu.edu.cn

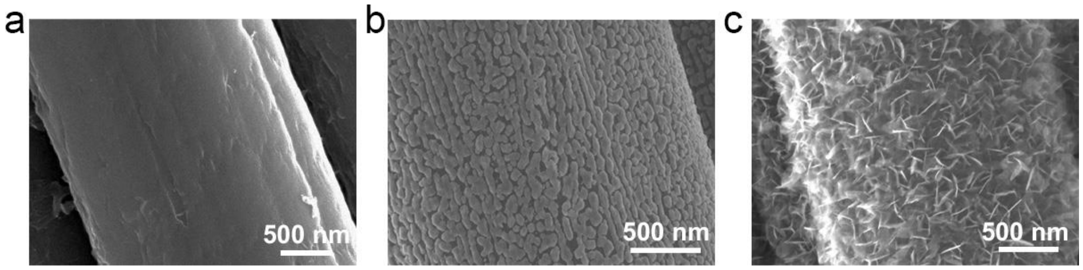

Figure S1. SEM images of (a) pristine CC, (b) R-SnO$_2$@CC and (c) F-SnO$_2$@CC.

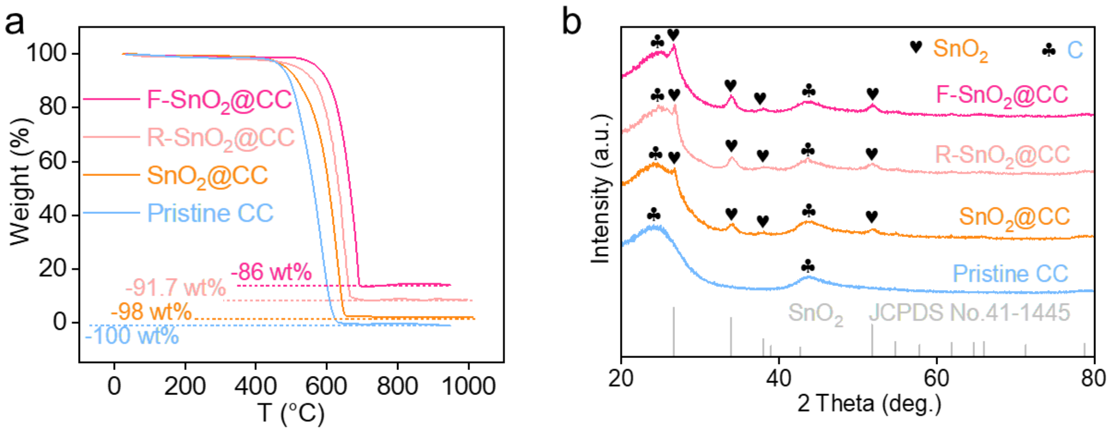

Figure S2. (a) TGA curves of pristine CC, SnO_2@CC, R-SnO_2@CC and F-SnO_2@CC. (b) XRD patterns of pristine CC, SnO_2@CC, R-SnO_2@CC and F-SnO_2@CC in comparison with the standard diffraction peaks for rutile SnO_2 (JCPDS No. 41-1445).

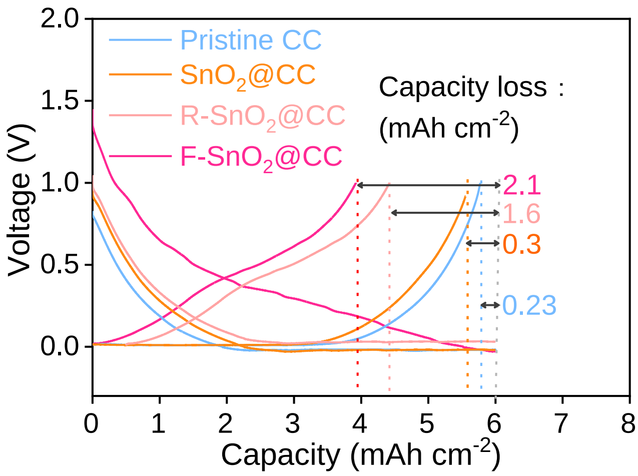

Figure S3. Capacity-voltage curves of pristine CC, SnO_2@CC, R-SnO_2@CC and F-SnO_2@CC electrodes plated at 0.5 mA cm^{-2} for 6 mAh cm^{-2} and stripped to 1 V cut-off voltage.

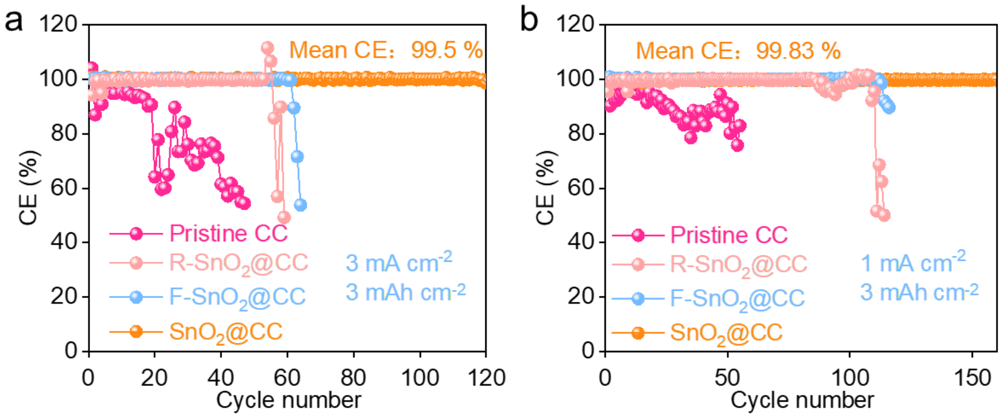

Figure S4. Coulombic efficiency of the pristine CC, SnO_2@CC, R-SnO_2@CC and F-SnO_2@CC electrodes at (a) 3 mA cm^{-2} for 3 mAh cm^{-2} and (b) 1 mA cm^{-2} for 3 mAh cm^{-2}.

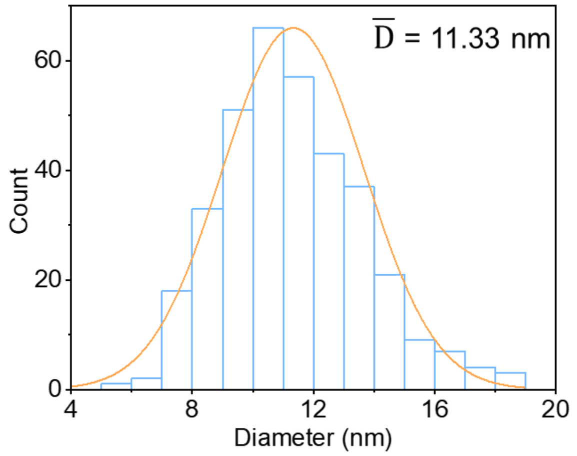

Figure S5. The particle size distribution curve of SnO_2 on CC.

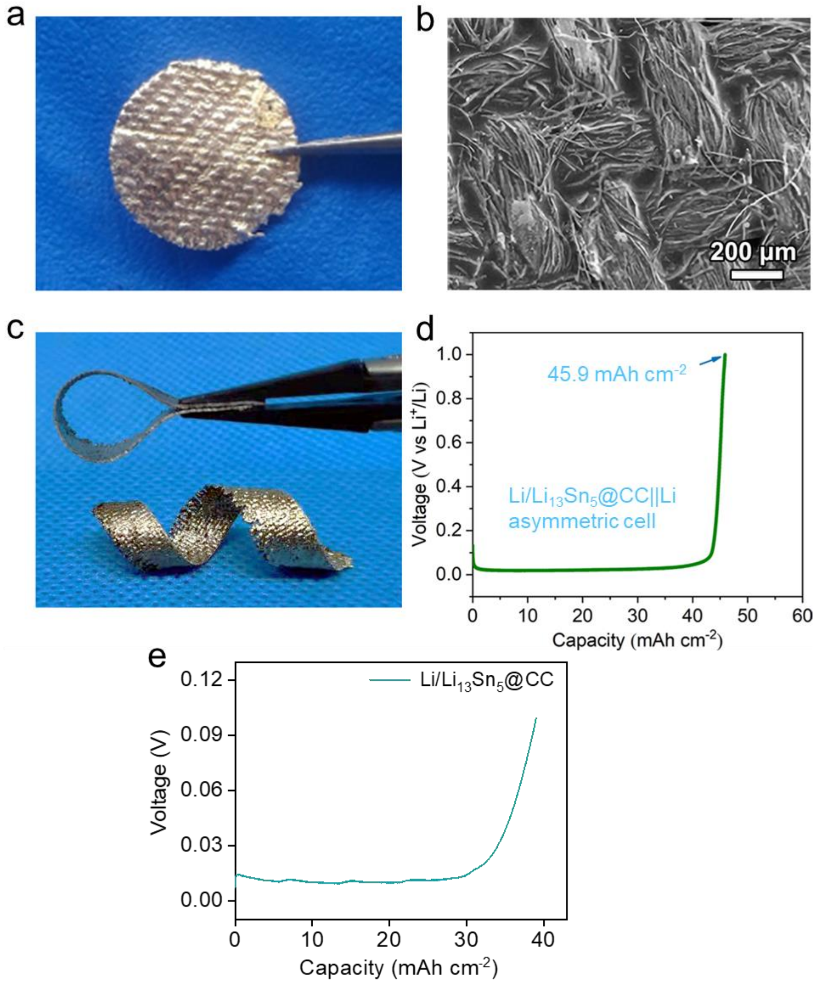

Figure S6. (a) Optical and (b) SEM images of $Li/Li_{13}Sn_5$@CC. (c) Optical images showing the twisting and bending of $Li/Li_{13}Sn_5$@CC. (d) Capacity-voltage curve of the $Li/Li_{13}Sn_5$@CC electrode after fully Li stripping. (e) Capacity-voltage curve of the $Li/Li_{13}Sn_5$@CC electrode after Li stripping to 0.1V.

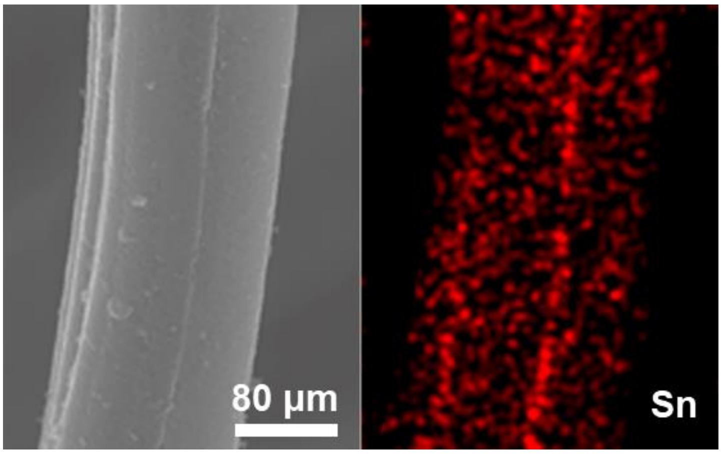

Figure S7. SEM and the Sn elemental mapping images of Li/$Li_{13}Sn_5$@CC electrode after Li stripping.

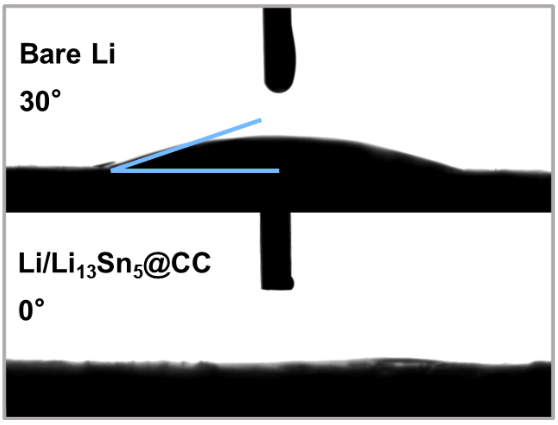

Figure S8. Contact angle test of bare Li and Li/$Li_{13}Sn_5$@CC electrode.

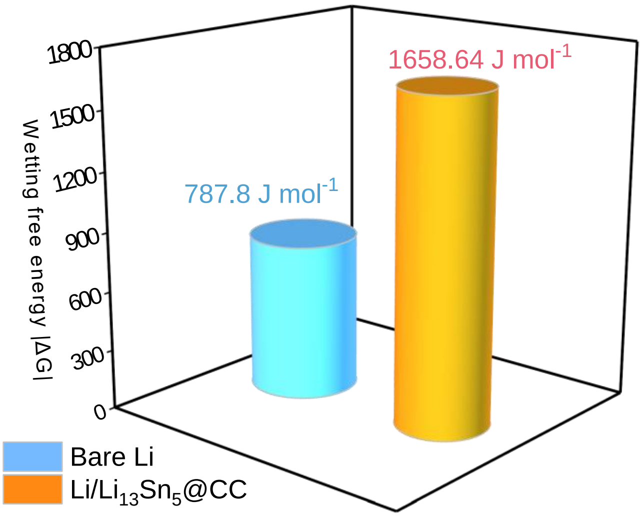

Figure S9. The wetting free energy of bare Li and Li/$Li_{13}Sn_5$@CC electrode.

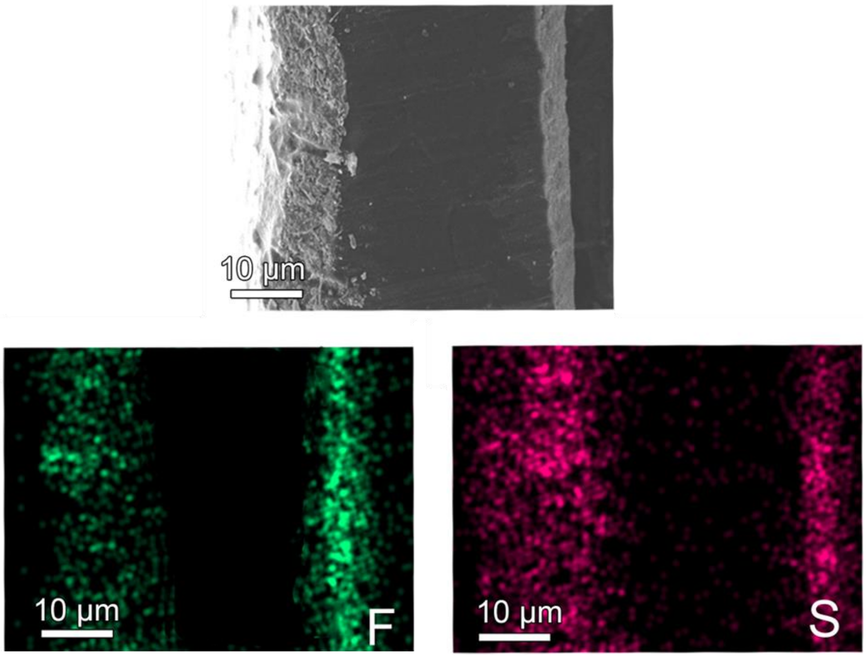

Figure S10. Cross-section SEM image of bare Li and its F and S elemental mappings.

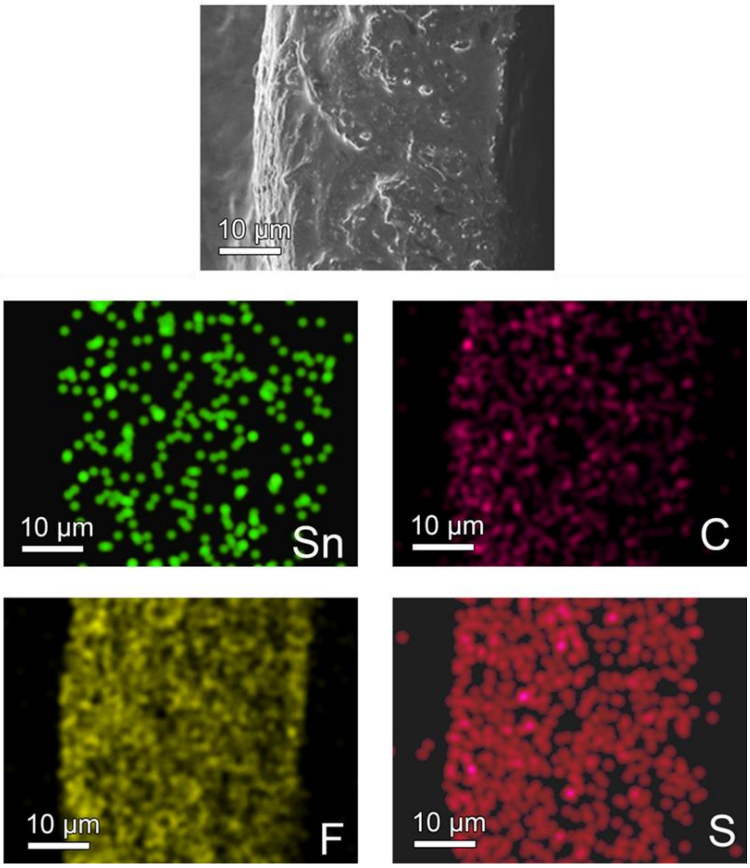

Figure S11. Cross-section SEM image of Li/$Li_{13}Sn_5$@CC and its Sn, C, F and S elemental mappings.

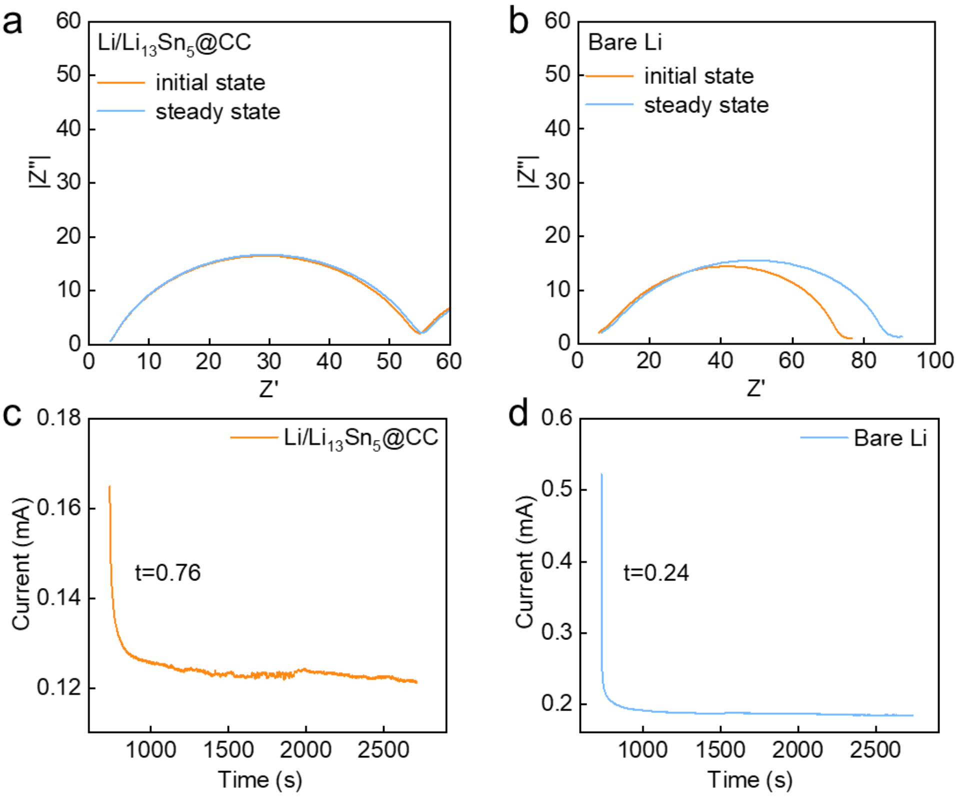

Figure S12. (a, b) EIS curves and (c, d) the associated current variation with time during polarization of $Li/Li_{13}Sn_5$@CC and bare Li symmetrical cells with applied potential difference of 10 mV.

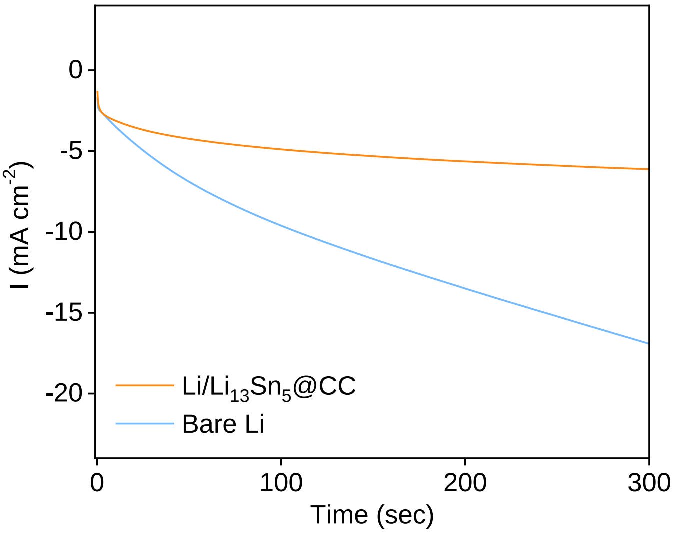

Figure S13. Chronoamperometry curves of $Li/Li_{13}Sn_5$@CC and bare Li at an overpotential of -150 mV.

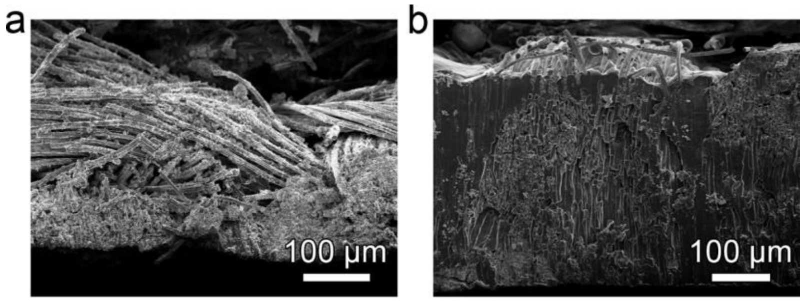

Figure S14. Cross-section SEM image of Li/$Li_{13}Sn_5$@CC after (a) 40 mAh cm^{-2} Li stripping and (b) 40 mAh cm^{-2} Li replating (87.1% DOD).

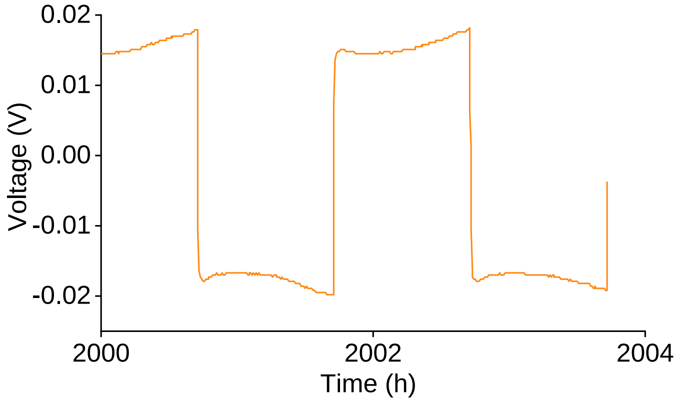

Figure S15. Enlarged voltage-time curves of cycling at 5 mA cm^{-2} for 5 mAh cm^{-2}.

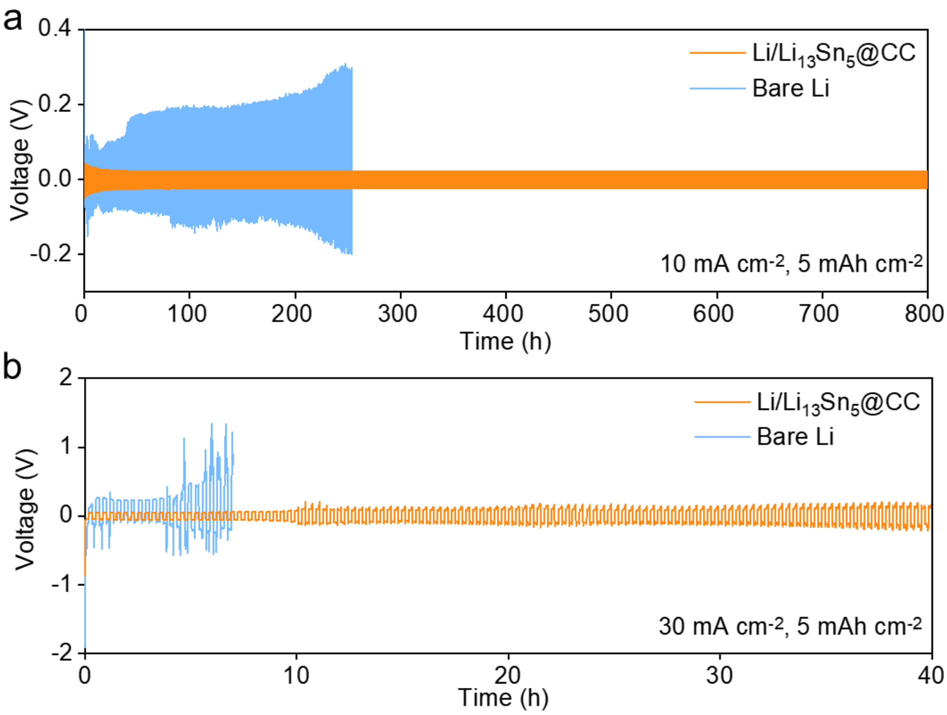

Figure S16. Cycling performance of Li/$Li_{13}Sn_5$@CC and bare Li symmetric cells at (a) 10 mA cm^{-2} and (b) 30 mA cm^{-2} with a fixed capacity of 5 mAh cm^{-2}.

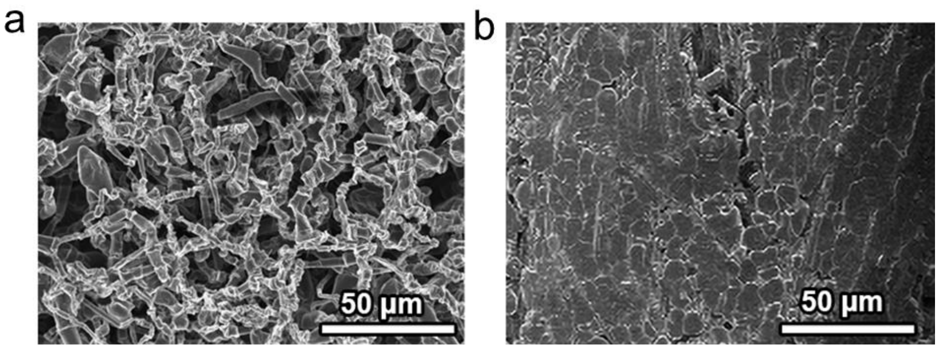

Figure S17. SEM images of (a) bare Li and (b) Li/$Li_{13}Sn_5$@CC after 50 cycles test at 30 mA cm^{-2} and 5 mAh cm^{-2}.

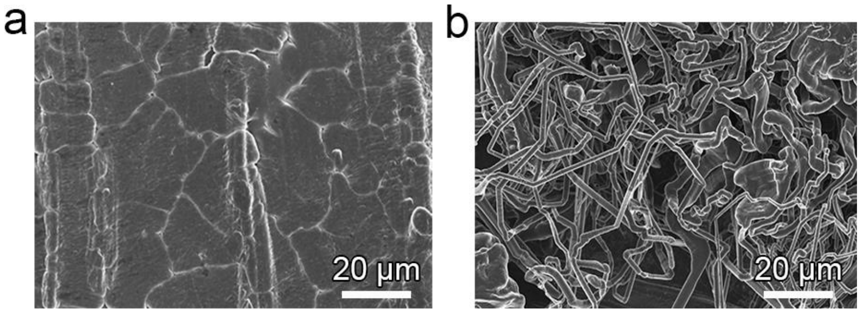

Figure S18. SEM images of (a) Li/$Li_{13}Sn_5$@CC after cycles of 2600 h and (b) bare Li after cycles of 700 h at 5 mA cm^{-2} and 5 mAh cm^{-2}.

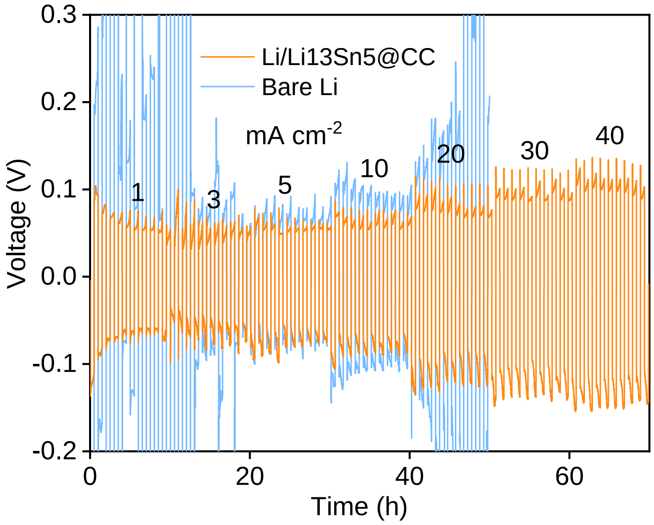

Figure S19. Rate performance of the symmetrical cells.

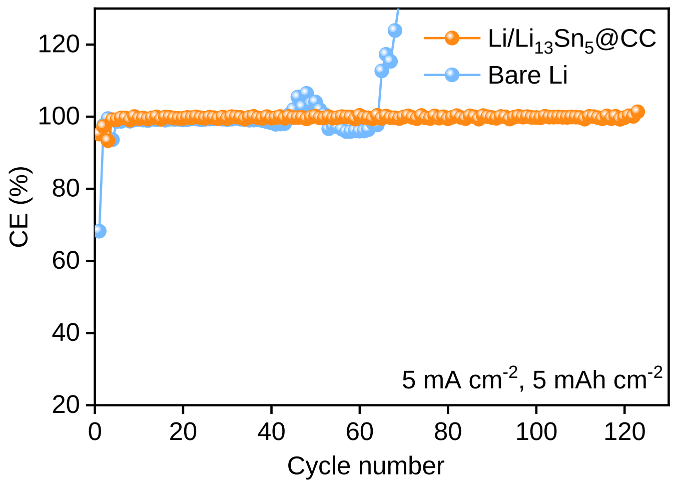

Figure S20. Coulombic efficiency of the Li/$Li_{13}Sn_5$@CC and bare Li electrodes at 5 mA cm^{-2} for 5 mAh cm^{-2}.

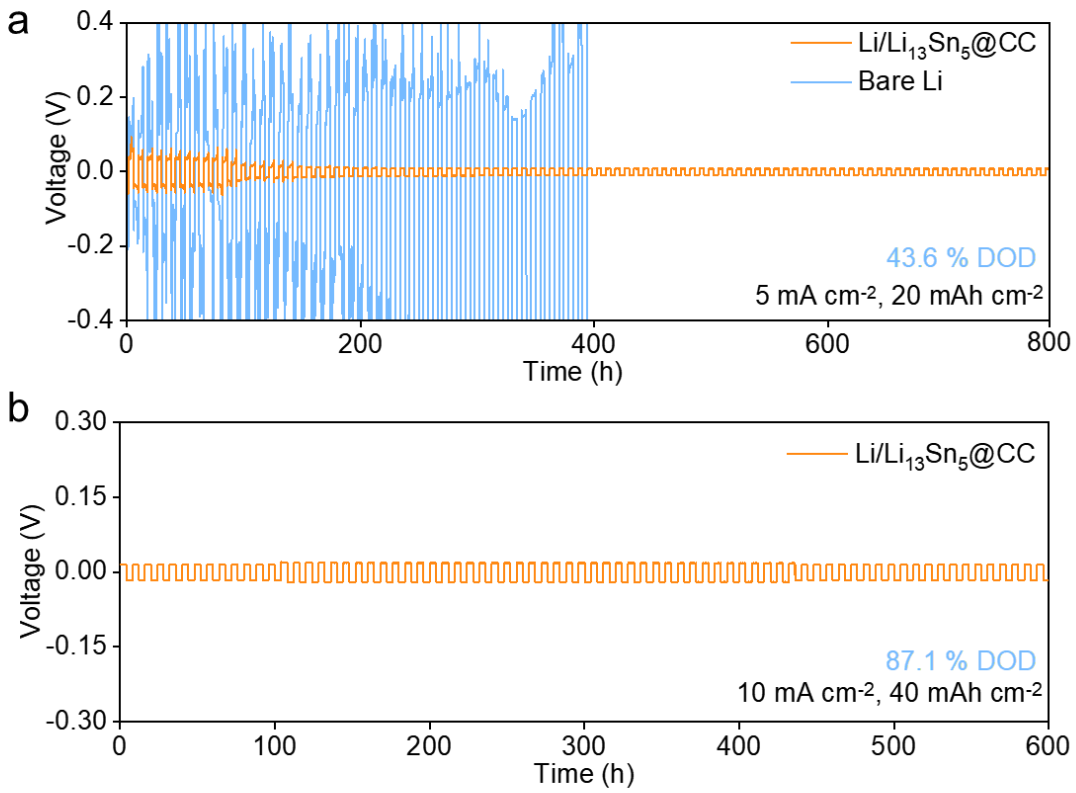

Figure S21. Cycling performance of Li/$Li_{13}Sn_5$@CC and bare Li symmetric cells at 5 mA cm^{-2} with a fixed capacity of (a) 20 mAh cm^{-2} and (b) 10 mA cm^{-2} with a fixed capacity of 40 mAh cm^{-2}.

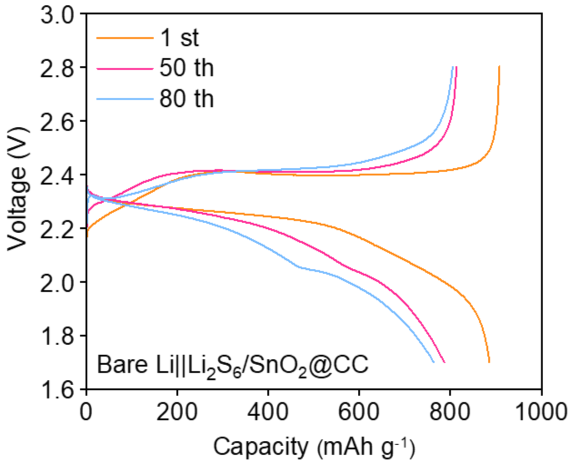

Figure S22. Charge-discharge voltage curves of bare Li||Li_2S_6/SnO_2@CC cell at different cycles.

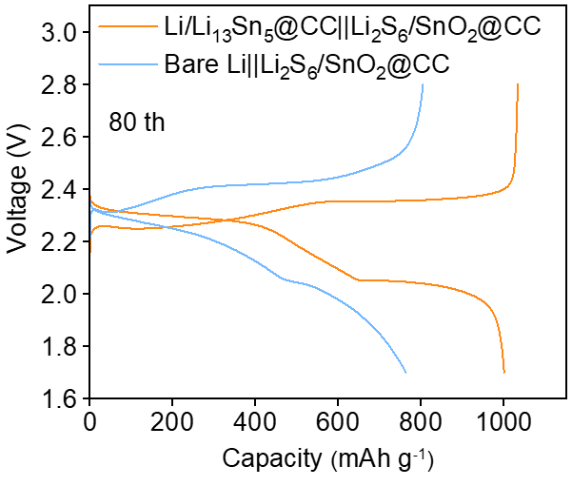

Figure S23. Comparison of Charge-discharge voltage curves of bare Li||Li_2S_6/SnO_2@CC and Li/$Li_{13}Sn_5$@CC||Li_2S_6/SnO_2@CC cells at 80th cycle.

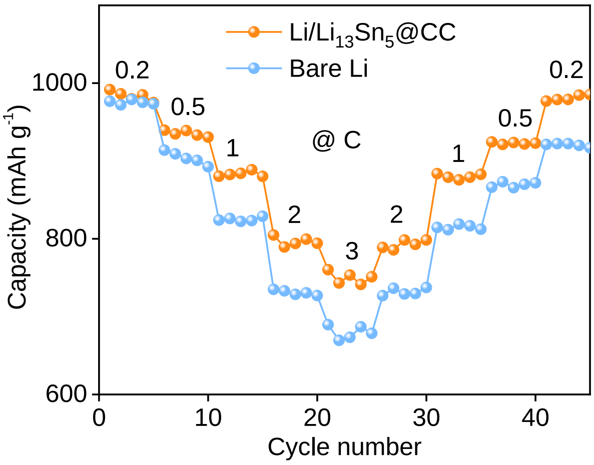

Figure S24. Rate capacities of Li/$Li_{13}Sn_5$@CC||Li_2S_6/SnO_2@CC and Li||Li_2S_6/SnO_2@CC batteries at various current rates from 0.2 C to 3 C.

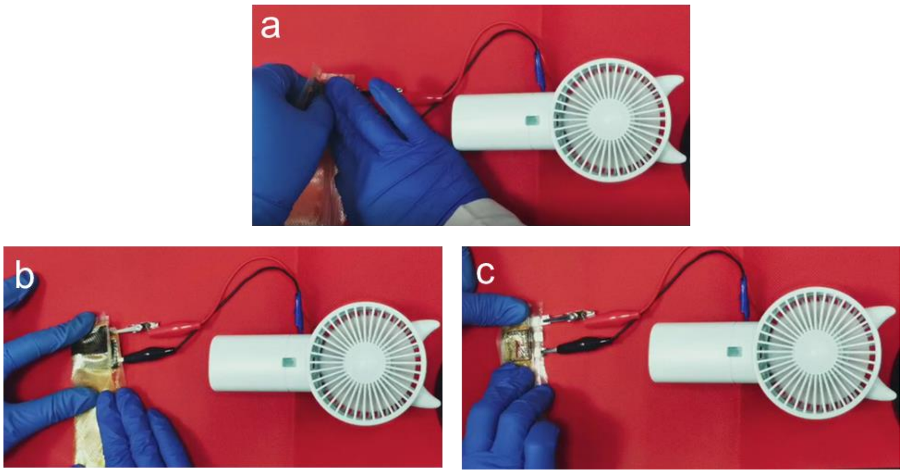

Figure S25. The Li/$Li_{13}Sn_5$@CC||Li_2S_6/SnO_2@CC pouch cell powering electric fans under (a) 90° bending, (b) one folding and (c) two folding states, respectively.

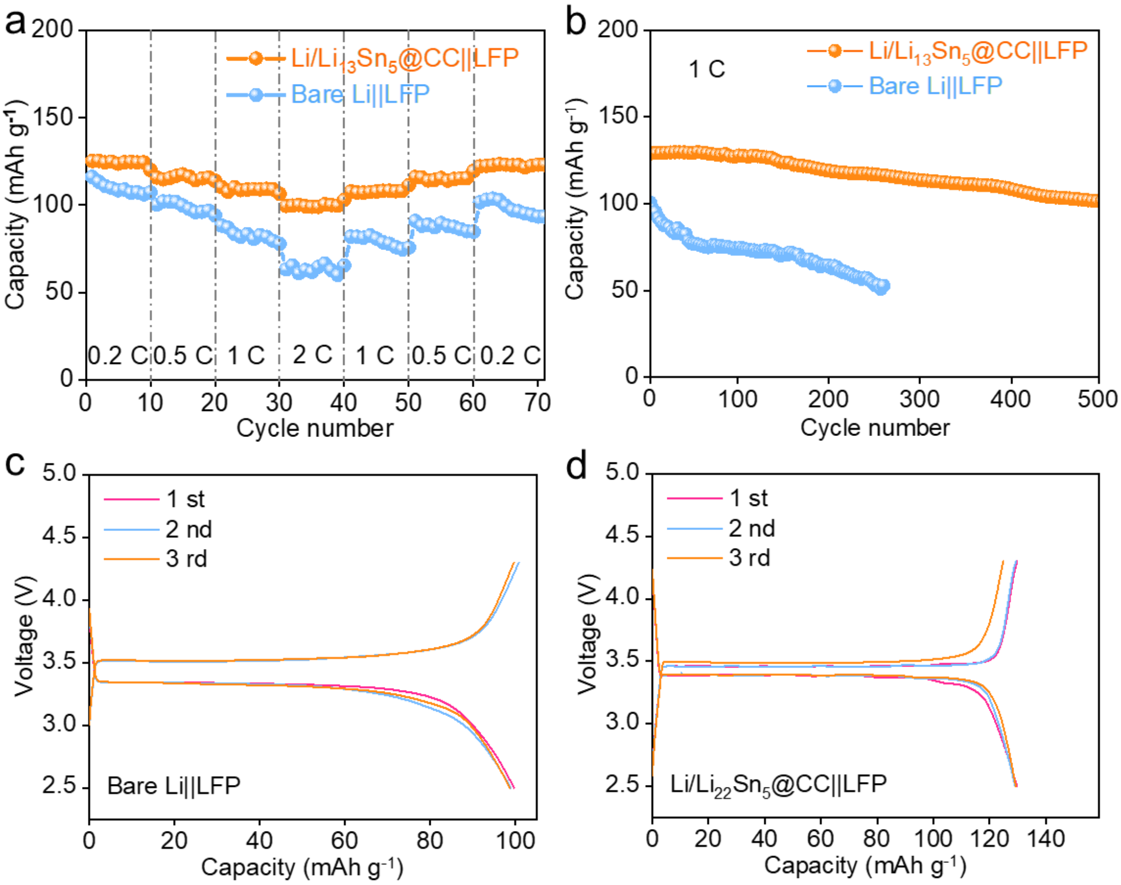

Figure S26. Electrochemical performance of full cell. (a) Rate performance. (b) Cycling performance at 1 C. Charge-discharge voltage curves of (c) bare Li||LFP full cell and (d) Li/$Li_{13}Sn_5$@CC||LFP full cell at different cycles.

Table S1. The actual weight change after molten Li infusion.

	**Pristine CC**	**SnO_2@CC**	**R-SnO_2@CC**	**F-SnO_2@CC**
**Pre-lithiation capacity (mAh cm^{-2})**	2.1	2.3	2.7	6
**Irreversible capacity (mAh cm^{-2})**	0.23	0.3	1.6	2.1

Table S2. Comparison of symmetric cell with the recently reported Sn-based LMAs.

**Types**	**Electrode**	**Current density (mA cm^{-2})**	**Areal capacity (mAh cm^{-2})**	**Cycles**	**Voltage hysteresis (mV)**	**Sn-containing (%)**	**Ref.**
**3D-host materials**	**Li/$Li_{13}Sn_5$@CC**	**5**	**5**	**1000**	**31.6**	**2**	**This work**
		**30**	**5**	**100**	**192**		
		**5**	**20**	**100**	**21**		
		**10**	**30**	**100**	**19.2**		
	Li-Li_xSn@CF	1	1	372	20	50	1
		3	1	180	160		
	Li-Li_xSn@CT	1	1	110	20	2.5	2
		3	1	55	40		
	Li-Li_xSn@CP	1	1	400	18	30	3
		2	1	250	20		
		5	1	100	120		
	Sn/C/Li	1	1	900	15	25	4
		2	1	1200	22		
		5	1	700	60		
	Li-Sn@NF	5	5	100	9.5	5	5
		10	5	200	27		
		3	1	300	100		
		10	1	1000	200		
	Li-Co@CS	1	1	400	50	/	6
		20	1	600	20		
		1	3	120	280		
	Li-Mn/G foam	1	1	800	30	/	7
		2	1	300	12		
	Li@Cu	1	1	800	12	/	8
		1	6	200	15		
		5	1	150	60		
	Li-N@CF	1	1	300	30	/	9
		3	1	270	50		
	Layered Li-rGO	1	1	100	100	/	10
		3	1	46	200		
**Protective layer**	Li_3Mg_7@Li	0.5	1	1000	11	/	11
		2	1	900	15		

	1	5	85	20		
PTMEG@Li	1	1	500	10	/	12
Li_xSn@Li	1	1	400	20	/	13
	3	1	150	50		
	5	1	250	100		
Li_2S@Li	5	2	937	20	/	14
LiF@Li	0.5	1	1000	150	/	15
	1	1	280	200		
	2	1	500	350		

Table S3. Comparison of Li/$Li_{13}Sn_5$@CC||Li_2S_6/SnO_2@CC||Li/$Li_{13}Sn_5$@CC cell under various deformation states with previously reported Li||S cells

Cell	S loading (mg cm^{-2})	Current density (mA cm^{-2})	Areal capacity (mAh cm^{-2})	Areal energy density (mWh cm^{-2})	Ref.
**Li/$Li_{13}Sn_5$@CC||Li_2S_6/SnO_2@CC**	**6.33**	**1.06**	**5.04**	**10.6**	**This work**
LV/Li-S	2	1.68	2.64	2.59	16
Li/CuCF- NSHG/S/NiCF	3.2	1	2	4.2	17
Li-HMSC	7.26	1	4	8.2	18
LiCSMF-S/CSMF	1.28	2	0.9	1.89	19
Li/CC-Graphene/S	5.1	4.27	4	8.2	20

Reference

[1] Y. Zhang, C. W. Wang, G. Pastel, Y. D. Kuang, H. Xie, Y. J. Li, B. Y. Liu, W. Luo, C. J. Chen, L. B. Hu, *Adv. Energy Mater.* **2018**, *8*, 1800635.

[2] W. S. Xiong, Y. Xia, Y. Jiang, Y. Qi, W. Sun, D. He, Y. Liu, X. Z. Zhao, *ACS Appl. Mater. Interfaces* **2018**, *10*, 21254.

[3] L. Tan, S. H. Feng, X. H. Li, Z. X. Wang, W. J. Peng, T. C. Liu, G. C. Yan, L. J. Li, F. X. Wu, J. X. Wang, *Chem. Eng. J.* **2020**, *394*, 124848.

[4] H. Qiu, T. Tang, M. Asif, W. Li, T. Zhang, Y. Hou, *Nano Energy* **2019**, *65*, 103989.

[5] Y. Xia, Y. Jiang, Y. Y. Qi, W. Q. Zhang, Y. Wang, S. F. Wang, Y. M. Liu, W. W. Sun, X. Z. Zhao, *J. Power Sources* **2019**, *442*, 227214.

[6] S. Li, Q. Liu, J. Zhou, T. Pan, L. Gao, W. Zhang, L. Fan, Y. Lu, *Adv. Funct. Mater.* **2019**, *29*, 1808847.

[7] B. Z. Yu, T. Tao, S. Mateti, S. G. Lu, Y. Chen, *Adv. Funct. Mater.* **2018**, *28*, 1803023.

[8] S. Huang, L. Chen, T. Wang, J. Hu, Q. Zhang, H. Zhang, C. Nan, L. Z. Fan, *Nano Lett.* **2021**, *21*, 791.

[9] L. Tao, A. Hu, Z. Yang, Z. Xu, C. E. Wall, A. R. Esker, Z. Zheng, F. Lin, *Adv. Funct. Mater.* **2020**, *30*, 2000585.

[10] D. Lin, Y. Liu, Z. Liang, H. W. Lee, J. Sun, H. Wang, K. Yan, J. Xie, Y. Cui, *Nat. Nanotechnol.* **2016**, 11, 626.

[11] H. Zhang, S. Ju, G. Xia, D. Sun, X. Yu, *Adv. Funct. Mater.* **2021**, *31*, 2009712.

[12] Z. Jiang, L. Jin, Z. Han, W. Hu, Z. Zeng, Y. Sun, J. Xie, *Angew. Chem. Int. Ed.* **2019**, *58*, 11374.

[13] S. Xia, X. Zhang, C. Liang, Y. Yu, W. Liu, *Energy Storage Mater.* **2020**, *24*, 329.

[14] H. Chen, A. Pei, D. C. Lin, J. Xie, A. K. Yang, J. W. Xu, K. X. Lin, J. Y. Wang, H. S. Wang, F. F. Shi, D. Boyle, Y. Cui, *Adv. Energy Mater.* **2019**, *9*, 1900858.

[15] Z. Peng, N. Zhao, Z. G. Zhang, H. Wan, H. Lin, M. Liu, C. Shen, H. Y. He, X. X. Guo, J. G. Zhang, D. Y. Wang, *Nano Energy* **2017**, *39*, 662.

[16] J. Wu, Z. Rao, X. Liu, Y. Shen, L. Yuan, Z. Li, X. Xie, Y. Huang, *Adv. Funct. Mater.* **2020**, *31*, 2009961.

[17] J. Chang, J. Shang, Y. Sun, L. K. Ono, D. Wang, Z. Ma, Q. Huang, D. Chen, G. Liu, Y. Cui, Y. Qi, Z. Zheng, *Nat. Commun.* **2018**, *9*, 4480.

[18] W. Xue, Z. Shi, L. Suo, C. Wang, Z. Wang, H. Wang, K. P. So, A. Maurano, D. Yu, Y. Chen, L. Qie, Z. Zhu, G. Xu, J. Kong, J. Li, *Nat. Energy* **2019**, *4*, 374.

[19] Z. Y. Wang, Z. X. Lu, W. Guo, Q. Luo, Y. H. Yin, X. B. Liu, Y. S. Li, B. Y. Xia, Z. P. Wu, *Adv. Mater.* **2021**, *33*, 2006702.

[20] B. Yu, Y. Fan, S. Mateti, D. Kim, C. Zhao, S. Lu, X. Liu, Q. Rong, T. Tao, K. K. Tanwar, X. Tan, S. C. Smith, Y. I. Chen, *ACS Nano* **2021**, *15*, 1358.
